# Embedding cognitive behavioural therapy for depression among patients with tuberculosis in Khyber Pakhtunkhwa, Pakistan: insights from a pilot trial process evaluation

**DOI:** 10.1136/bmjopen-2025-115224

**Published:** 2026-11-09

**Authors:** Fayaz Ahmad, Aiman Niaz, Fatima Khalid Qazi, Shaista Rasool, Rubab Jawed, Asad Ullah, Nishani Fonseka, Saima Shiekh, Zohaib khan, Saeed Farooq

**Affiliations:** 1 Institute of Public Health & Social Sciences, Khyber Medical University, Peshawar, Pakistan; 2 Office of Research, Innovation & Commercialization (ORIC), Khyber Medical University, Peshawar, Pakistan; 3 School of Medicine, Keele University, Keele, UK; 4 Department of Rheumatology and Musculoskeletal Sciences (NDORMs), University of Oxford, Oxford, UK

**Keywords:** PUBLIC HEALTH, Depression, Postpartum, Implementation Science, Tuberculosis, QUALITATIVE RESEARCH

## Abstract

**Abstract:**

**Objectives:**

To assess the acceptability and feasibility of a cognitive behavioural therapy (CBT) intervention for depression in tuberculosis (TB) care in Pakistan and to identify contextual factors influencing its implementation. We also evaluated training for TB health workers (TBHWs) and refined the intervention and trial procedures.

**Design:**

A mixed-methods process evaluation was conducted concurrently with the in-built pilot trial.

**Setting:**

The COgNitive Therapy for depRessiOn in TubercuLosis (CONTROL) intervention, comprising CBT therapy, was delivered at four TB Basic Management Units that provide drug-sensitive TB services and are located within primary, secondary and tertiary healthcare facilities in two districts of Khyber Pakhtunkhwa Province, Pakistan: Peshawar and Haripur. The semi-structured interviews (SSIs) and fidelity assessments were conducted on-site, while training and focus group discussions (FGDs) were held at Khyber Medical University, Peshawar, Pakistan.

**Participants:**

Quantitative data examined recruitment, retention, follow-up completion, therapist competency and training feedback. Qualitative data from SSIs (n=15) and FGDs (n=3) with patients, professionals who delivered CBT, medical officers (MOs) and trial staff were analysed thematically using a framework approach.

**Primary and secondary outcome measures:**

Acceptability, feasibility, contextual influencing factors of intervention and trial procedures, including intervention refinement and TBHW training effectiveness.

**Results:**

Qualitative and quantitative findings converged across the Medical Research Council (MRC) framework. 1). Implementation showed that recruitment, retention, intervention fidelity and therapist competency were high, and training was positively received. Qualitative findings supported feasibility while identifying workload, assessment burden, space availability and patient literacy as implementation challenges requiring adaptation. 2). Mechanisms of impact showed strong therapist competency in interpersonal effectiveness, collaboration, guided discovery and feedback converged with reports of therapeutic alliance and open communication. Competent delivery of CBT techniques was accompanied by perceived improvements in emotional well-being, motivation, treatment adherence and interpersonal relationships. 3). Context: language, literacy, age and access to digital resources influenced patient understanding and engagement. Patient-centred communication and visual materials improved comprehension, while limited mobile-phone access, workload, scheduling and intervention resources challenged delivery and continuity of care.

**Conclusions:**

The findings support the feasibility and acceptability of integrating CBT into routine TB care. Insights from this evaluation guided the refinement of the CBT intervention and implementation strategy for the definitive CONTROL trial. Future work should assess the intervention’s effectiveness, scalability and cost-effectiveness.

**STRENGTHS AND LIMITATIONS OF THIS STUDY**

- Methodological triangulation, combining qualitative and quantitative data from therapists (directly observed treatment short facilitators), medical officers, trial staff and patients, enhanced the rigour and credibility of the findings by capturing convergent perspectives from both the intervention team and its recipients.
- Fidelity was assessed using the Revised Cognitive Therapy Scale, a validated cognitive behavioural therapy (CBT) competency measure, against a pre-specified competency threshold, providing an objective, standardised measure of intervention delivery quality.
- The qualitative sample was limited to patients who attended at least four CBT sessions; those who discontinued earlier were not interviewed, which may have under-represented less favourable experiences of the intervention.
- As a process evaluation within a pilot randomised controlled trial, it was not designed to examine cost-effectiveness, so the economic implications of the intervention remain unassessed. Fidelity was assessed through direct structured observation rather than audio or video recording, which may have missed some real-time protocol deviations; the presence of an observer may also have introduced a Hawthorne effect, with therapists adhering more closely to the protocol than they would in routine, unobserved practice.
- The definitive COgNitive Therapy for depRessiOn in TubercuLosis trial will address these limitations through audio-recorded fidelity assessment, broader qualitative sampling irrespective of session attendance and a planned cost-effectiveness analysis.

## Introduction

 Tuberculosis (TB) remains the leading cause of death from a single infectious agent worldwide, even surpassing HIV in mortality rates.^1^ Despite being preventable and curable, around one-third of the world gets infected with TB, contributing to nearly 1.6 million deaths annually.^2^ More than 95% of TB-related deaths occur in low- and middle-income countries (LMICs), where barriers such as limited healthcare access, malnutrition and substandard living conditions contribute to increased infection rates and delayed treatment.^3 4^ Pakistan ranks fifth among high-burden countries for TB globally, with an estimated 510 000 new cases of TB each year, of which approximately 15 000 present with drug-resistant strains.^5^

Depression is a common comorbidity in TB, with studies documenting its prevalence to be three to six times higher in patients with TB than in the general population.^6^ TB and depression form a syndemic relationship, with bidirectional interactions that mutually intensify each condition via biological, social and behavioural pathways.^7 8^ TB may lead to depression, as it disrupts serotonin production, and some anti-TB medications have been linked to the onset of depression symptoms.^9^ Moreover, the experience of living with TB is marked by stigma and social isolation, which can contribute to psychological distress.^10^ A study from Pakistan found that nearly half of the patients with TB were suffering from depression.^11^ Depression in patients with TB is associated with poor medication adherence and non-compliance with the treatment regimen, consequently increasing the likelihood of drug resistance, death and continued transmission of disease within the community.^8^

Evidence suggests that integrating psychological support into TB care enhances treatment adherence, reduces patient attrition, improves quality of life and enhances depression management.^12 13^ The End TB strategy of the WHO calls for integrating mental health services into TB care frameworks, emphasising the need for comprehensive care models that address both conditions simultaneously.^14^ In many LMICs, including Pakistan, TB treatment services are well integrated across primary, secondary and tertiary levels of care.^15^ However, Pakistan’s national TB programme currently lacks structured guidance for integrating mental health support, particularly for identifying and managing depression among patients with TB. Furthermore, there are limited evidence-informed or standardised care packages addressing depression within TB care in the context of Pakistan.^16^

Cognitive behavioural therapy (CBT) is the preferred non-pharmacological treatment for addressing depression.^17^ It operates by targeting negative thought patterns and behaviours, strengthening emotional regulation and improving problem-solving skills.^18^ Beyond depression, it is effective in addressing the psychological management of chronic diseases, such as asthma, diabetes and chronic obstructive pulmonary disease and also chronic infectious diseases like TB.^17 19^ Nevertheless, for CBT to achieve its intended outcomes, the therapist must be trained to demonstrate expertise and understanding in their interactions with patients.^20^ However, in many LMICs, there is a significant shortage of specialised mental health professionals. As a result, psychological therapies are being more frequently provided by non-specialist healthcare providers, healthcare providers with no training in mental health specialties.^21 22^ This practice reflects a broader health systems approach known as task shifting, which has gained traction as a practical and cost-effective strategy for addressing healthcare needs. In essence, task shifting involves reallocating specific responsibilities from specialist health professionals to health workers with shorter training and fewer qualifications.^23^

The COgNitive Therapy for depRessiOn in TubercuLosis treatment (CONTROL) is a research programme aimed at improving depression and enhancing TB treatment outcomes in people with drug-sensitive TB (DSTB) and multidrug-resistant TB.^24^ The programme includes four work packages: 1) development of a CBT-based intervention (CONTROL intervention), 2) an in-built pilot trial, 3) a pragmatic, parallel-arm randomised controlled trial (RCT) following a hybrid type-1 implementation approach and 4) developing a strategy for the scale-up using the theory of behaviour change. The CONTROL intervention was developed in the first stage of the research programme, using evidence synthesis, ethnographic and qualitative methods and incorporating perspectives from patients, caregivers and healthcare providers. Once the intervention was developed, a pilot trial was conducted before a large-scale trial to assess its real-world implementation and methodological robustness.^24^ This approach enables the early identification and correction of potential issues, thereby optimising the main trial’s design and resource efficiency. Further, to inform progression to the definitive trial, an embedded process evaluation was conducted alongside the pilot trial.

Although clinical trials evaluate whether an intervention is effective, they provide limited understanding of the processes through which interventions are delivered and embedded in routine practice. Therefore, process evaluations conducted alongside or after trials are essential for examining implementation, participants’ and providers’ experiences and the contextual factors that influence intervention delivery and trial outcomes.^25^ The Medical Research Council (MRC) framework for evaluating complex interventions provides structured guidance for such evaluations, emphasising three core dimensions: implementation, mechanisms of impact and context across four iterative stages: development, feasibility/piloting, evaluation and implementation.^26^ Although process evaluation can be conducted throughout all stages of developing and evaluating complex interventions, in this study, it is primarily aligned with the feasibility and piloting stage of the MRC framework. At this stage, it helps determine whether the intervention and trial procedures can be implemented as intended, identifies contextual influences on delivery and informs refinement of the intervention and trial procedures before progression to a definitive trial.

Before progressing to the definitive CONTROL trial, several important implementation and trial uncertainties remained. It was unclear whether trained TB health workers (TBHWs) could deliver the task-shifted CBT intervention with adequate fidelity, whether the intervention would be acceptable and practical within routine TB services and which contextual factors might influence its implementation. In addition, uncertainties remained regarding the feasibility of participant recruitment, retention, follow-up and delivery of training within routine TB healthcare settings. The pilot process evaluation was therefore embedded to examine these implementation processes and outcomes, identify contextual factors requiring refinement and optimise both the intervention and trial procedures before progression to the definitive trial.

## Methods

An in-built pilot trial with embedded process evaluation was conducted in four TB centres in Khyber Pakhtunkhwa (KP), Pakistan, to assess the acceptability and feasibility of implementing CBT within the operational context of the Provincial Tuberculosis Control Programme (PTP) in KP. In PTP, TBHWs, who serve as Directly Observed Treatment Short-course (DOTS) facilitators (referred to as therapists in this study) and provide routine TB care, were trained to deliver CBT to patients with TB. Patients in the intervention arm received six sessions of CBT, each lasting 40 to 60 min, delivered by a therapist. The use of a flipbook supported the delivery of the intervention, along with videos for facilitators, while audios and homework for patients. Patients also received Enhanced Treatment as Usual (ETAU) from medical officers (MOs) trained in the Mental Health Gap Action Programme (mhGAP), who worked in the same TB centres.^24^ Comprehensive details of the CONTROL intervention and trial design are available in the published study protocol.^24^

### Study design

This process evaluation employed a mixed-methods approach, integrating both qualitative and quantitative components. We used the UK’s MRC framework^27^ to conduct a rigorous process evaluation of the CBT intervention piloted within the CONTROL pilot trial.^24^

### Study settings

The CONTROL intervention, comprising CBT therapy, was delivered at four TB Basic Management Units that provide DSTB services and are located within primary, secondary and tertiary healthcare facilities in two districts of KP Province, Pakistan: Peshawar and Haripur. The semi-structured interviews (SSIs) and fidelity assessments were conducted on-site, whereas the trainings and focus group discussions (FGDs) were held at Khyber Medical University, Peshawar, Pakistan.

### Study participants and sampling

#### Qualitative component

Participants for the qualitative SSIs and FGDs were purposively selected to capture diverse perspectives from key stakeholders involved in the implementation and receipt of the CBT intervention, including patients, therapists (DOTS facilitators), MOs and trial staff. A total of 15 SSIs and 3 FGDs were conducted (table 1). Three FGDs were conducted, one with each predefined stakeholder group (MOs, therapists and trial staff), to capture collective perspectives within these groups. The sample size for SSIs was guided by the need to capture sufficient variation and information across the stakeholder groups rather than by a predetermined number. Data collection for SSIs continued until thematic saturation was achieved, whereby no new codes or themes emerged from successive interviews and discussions.

**Table 1:** Details of qualitative interviews, participant groups and timeline

Group of participants	SSIs	FGDs	Timeline
Patients who completed the intervention (attended five or all six sessions)	5	Nil	After 8 weeks post-randomisation
Patients who could not complete the intervention (attended 1–4 sessions for maximum variation in perspectives)	0	Nil	After 8 weeks post-randomisation
Patients who completed the intervention (attended five or all six sessions)	2	Nil	After 24 weeks post-randomisation
Patients who completed the intervention (attended five or all six sessions)	2	Nil	After 32 weeks post-randomisation
Patients in the control arm	5	Nil	After 24 weeks post-randomisation
Trial coordinator	1	Nil	After 32 weeks post-randomisation
Therapists (DOTS facilitators)	Nil	1	After the delivery of the final session to all the trial participants
Medical officers	Nil	1	After 24 weeks post-randomisation
Trial staff	Nil	1	After 32 weeks post-randomisation
**Total**	15	3	

DOTS, directly observed treatment short; FGDs, focus group discussions; MO, medical officers; SSIs, semi-structured interviews.

Among the SSIs, we conducted five interviews with patients who completed the intervention (attended at least five out of a total of six CBT sessions) at 8 weeks post-randomisation, two at 24 weeks and two at 32 weeks (table 1). We also planned to interview patients who could not complete the intervention to capture a range of experiences, including partial exposure to the intervention. However, we could not conduct any. Only two eligible patients were identified: one declined participation due to a recent carcinoma diagnosis, and the other had relocated out of the city and could not be reached for an interview. An additional five interviews were conducted with patients in the control arm at 24 weeks post-randomisation. One interview was also conducted with the trial coordinator to understand the entire pilot process.

One FGD was conducted with therapists (DOTS facilitators) after the final intervention session, with MOs at 24 weeks post-randomisation and with trial staff at 32 weeks post-randomisation. The timing of the interviews was aligned with key trial milestones. Patient interviews were conducted after intervention completion and follow-up assessments to capture both immediate and longer-term experiences, while interviews and FGDs with therapists, MOs and trial staff were conducted after they had completed their respective roles in the pilot trial to facilitate comprehensive reflection on the implementation process.

#### Quantitative component

The quantitative component primarily assessed the feasibility of trial procedures, feedback on training and the fidelity of intervention. The feasibility parameters were predefined to determine whether the pilot trial procedures were practical and acceptable and to inform progression to the definitive trial. These parameters, guided by the pre-specified traffic light criteria,^24^ included recruitment, retention in therapy sessions and follow-up completion. Recruitment was considered feasible if a target sample of 80 participants could be enrolled within 3 months. Retention in therapy was operationalised as the proportion of participants who attended at least four intervention sessions, with a threshold of 70% indicating acceptable retention. Follow-up completion was evaluated by the proportion of participants completing the 24-week follow-up assessment, and feasibility was defined as achieving a follow-up rate of at least 75%. The feasibility of engaging TBHWs was assessed by the number approached and the number who successfully completed the training programme in both the intervention and ETAU arms. Therapists were asked to complete training feedback forms to assess their satisfaction with the content, delivery and relevance of the training.

For fidelity assessment, 10% of the total intervention sessions were selected using a random sampling technique.^28–30^ The intervention consisted of six CBT sessions, expected to be delivered to 40 participants in the intervention arm, for a total of 240 sessions. A computer-generated randomisation procedure was used to select 24 sessions (10% of the total) for fidelity assessment. These sessions were proportionally distributed, with four sessions randomly chosen for each of the six CBT session types. This random sampling ensured an unbiased evaluation of intervention delivery and therapist competence.^28^

### Data collection procedure

Data for the process evaluation were collected from May to December 2024, concurrently with the pilot trial (table 1).

#### Qualitative data

We developed separate topic guides for each participant’s group, which were translated into the local languages (Urdu and Pashto). Each topic guide was developed in accordance with the MRC framework^31^ to explore key implementation outcomes, including feasibility and acceptability, as well as identifying contextual barriers and facilitators. Moreover, the guides sought to elicit participant perspectives on potential adaptations to optimise the intervention for the definitive trial. All topic guides were reviewed and validated by a multidisciplinary team comprising a qualitative research expert, a public health specialist, clinical psychologists and members of the research user group before data collection began. FGDs lasted 50–70 min, and SSIs lasted 30–45 min. The durations of interviews for SSIs and FGDs were considered appropriate to explore participants’ experiences and perceptions in depth while minimising participant burden and accommodating the practical constraints of working in routine TB service delivery. To acknowledge participants’ time and contribution, a cash reimbursement of 2800 PKR (approximately 7.5 GBP) was provided.

SSIs and FGDs were conducted by a research team trained in qualitative research methods and qualitative data collection. All participants were provided with an information sheet detailing the purpose and procedures of the interview before the interview commenced. Written informed consent was obtained from each participant for both participation in the study and audio recording of the SSIs and FGDs. All the interviews were transcribed verbatim, translated into English, anonymised and proofread against the audio recordings by the research team.

#### Quantitative data

Quantitative data related to key feasibility indicators, including participant recruitment, retention in therapy and completion of follow-up assessments, were systematically recorded throughout the pilot trial. The field team maintained detailed logs in structured Excel sheets, with daily entries documenting trial activities. These logs served as the primary data source for evaluating feasibility outcomes and were reviewed and analysed after the pilot phase.

Regarding therapist training, participant attendance was systematically recorded to monitor engagement throughout the training days. On the final day, trainees completed a structured feedback form comprising nine items. To facilitate comprehension among all participants, the form was translated into Urdu.

The fidelity of the CBT sessions was assessed using two standardised observation tools: the CBT Assessment Checklist and the Revised Cognitive Therapy Scale (CTS-R). Fidelity to the intervention content was evaluated using a structured checklist (online supplemental file A), with each component rated on a 5-point Likert scale from 0 (‘not implemented’) to 4 (‘fully implemented’). This session-specific CBT assessment checklist was developed for each of the six CBT sessions and underwent expert review by trained CBT professionals to ensure content validity. Similarly, the CTS-R,^32^ a validated 12-item instrument, was employed to assess therapist competence and fidelity to the CBT model. CTS-R (online supplemental file B) covers 12 domains rated on a 7-point Likert scale (0=incompetent to 6=expert). The maximum score is 72, with the Newcastle Cognitive Therapy Centre defining a minimum competence threshold of 36,^32 33^ corresponding to an average score of 3 per domain. Both content adherence and therapist competence metrics were analysed to provide a comprehensive assessment of intervention fidelity.

These assessments were conducted by trained master trainers, psychologists holding diplomas in CBT, who systematically evaluated the delivery of sessions against predefined criteria. Master trainers observed each CBT session in its entirety and used fidelity checklists to systematically assess its delivery. Before any observation, written informed consent was obtained from both therapists and patients, ensuring ethical compliance for session assessment.

### Data analysis

#### Qualitative data

We employed a framework analysis approach using a mix of deductive and inductive techniques. We first developed a thematic framework based on the research questions, topic guides, thematic analysis of five transcripts and the MRC framework. The data were then analysed using the framework, with a focus on perceptions of intervention and implementation challenges. The thematic framework was systematically applied to the transcribed data, with summaries of participants’ responses and verbatim quotes. The charted data were reviewed and analysed to compare views, seeking patterns, connections and explanations. After several iterations and cross-checks of data among the analysis team members, summary findings were finalised for each participant group. Finally, we integrated the key findings from the four participant groups and explored their similarities and differences.

#### Quantitative data

Feasibility indicators were analysed descriptively using percentage distributions. These metrics were evaluated against the predefined feasibility cut-off points specified in the study protocol^24^ to determine whether the pilot trial met the anticipated benchmarks. Similarly, training attendance was documented, and the training feedback data were analysed descriptively, with response frequencies expressed as percentages.

A fidelity assessment was conducted to measure adherence to the intervention protocol. Frequencies and proportions were calculated for each checklist item across all observed sessions. Session-level fidelity scores were obtained by summing ratings of individual components, and overall fidelity was determined by averaging the scores and calculating SD across the randomly selected CBT sessions.

### Data triangulation

We employed methodological triangulation,^34^ combining qualitative and quantitative findings to generate a comprehensive understanding of the intervention’s implementation. Triangulation occurred at two levels: first, through data source triangulation by comparing qualitative findings across different participant groups (patients, therapists (DOTS facilitators), MOs and trial staff) to identify converging and contrasting perspectives; and second, by using joint display to integrate quantitative and qualitative findings within the MRC process evaluation domains, facilitating comparison and interpretation of evidence across both datasets. This triangulation enhanced the interpretive depth, credibility and comprehensiveness of the process evaluation.

### Patient and public involvement

Patient and public involvement and engagement (PPIE) was integrated throughout this mixed-methods process evaluation to ensure its relevance and acceptability within the trial context. Before data collection, a PPIE meeting was held with a service-user group comprising patients and healthcare providers, who reviewed the draft data collection tools and participant-facing materials. Their feedback led to key refinements, including simplifying language for clarity, scheduling interviews and focus groups at times convenient for participants and conducting group discussions within university settings to maximise comfort and accessibility. These contributions improved the cultural appropriateness, feasibility and overall quality of the process evaluation.

## Results

### Demographics

A total of 79 participants took part in the CONTROL pilot trial. The average age was 37.49 years (SD±1.99). Of these, 31 participants (39.24%) were male, and 48 participants (60.76%) were female. Most participants were Pakistani nationals (65 individuals, 82.28%), while 14 participants (17.72%) were Afghan nationals. The demographics of the interview participants are presented in table 2 below.

**Table 2:** Demographic characteristics of the qualitative sample

Patients		
Characteristic	Intervention arm (n=9)	Control arm (n=5)
Gender	Male: 6 (66.7%); female: 3 (33.3%)	Male: 2 (40.0%); female: 3 (60.0%)
Age (years)	Mean: 37.1 (SD: 18.7); range: 18–66	Mean: 39.6 (SD: 22.5); range: 18–75
Marital status	Married: 5 (55.6%); unmarried: 4 (44.4%)	Married: 4 (80.0%); unmarried: 1 (20.0%)
Nationality	Pakistani: 7 (77.8%); Afghan: 2 (22.2%)	Pakistani: 5 (100%)
Education	Uneducated: 4 (44.4%); educated: 5 (55.6%)	Uneducated: 4 (80.0%); educated: 1 (20.0%)
Employment	Unemployed: 4 (44.4%); employed: 5 (55.4%)	Unemployed: 3 (60.0%); employed: 2 (40.0%)
Facility area	Peshawar: 6 (66.7%); Haripur: 3 (33.3%)	Peshawar: 3 (60.0%); Haripur: 2 (40.0%)
**Characteristic**	**Trial team (n=14**)	**Health professionals (n=8**)
Gender	Female: 10 (66.7%); male: 5 (33.3%)	Male: 8 (100%)
Age (years)	Mean: 28.3 (SD: 5.4) Range: 24–44	Mean: 51.0 (SD: 10.1) Range: 30–59
Designation	Research assistants: 6 (40%); psychologists: 4 (26.7%); site coordinators: 4 (26.7.%) Trial coordinator 1 (6.6%)	Medical officers: 4 (50.0%); DOTS facilitators: 4 (50.0%)
Experience (years)	Mean: 4.6 (SD: 2.4); range: 1–10	Mean: 25.8 (SD: 10.6), Range: 7–38
Facility area	Peshawar: 10 (71.4%); Haripur: 4 (28.6%)	Peshawar: 6 (75.0%); Haripur: 2 (25.0%)

DOTS, directly observed treatment short; MO, medical officers.

The findings are organised according to the three MRC process evaluation domains: 1) implementation, 2) mechanism of impact and 3) context of the CONTROL intervention. Within each domain, quantitative and qualitative findings are integrated to provide a comprehensive understanding of the feasibility, acceptability, fidelity and contextual influences on the implementation of the CONTROL intervention. Figure 1 presents the conceptual framework developed from the process evaluation findings, illustrating how the integrated qualitative and quantitative findings map onto the three core domains of the MRC process evaluation framework. Illustrative participant quotations supporting the qualitative findings are presented in online supplemental table 1.

**Figure 1:**
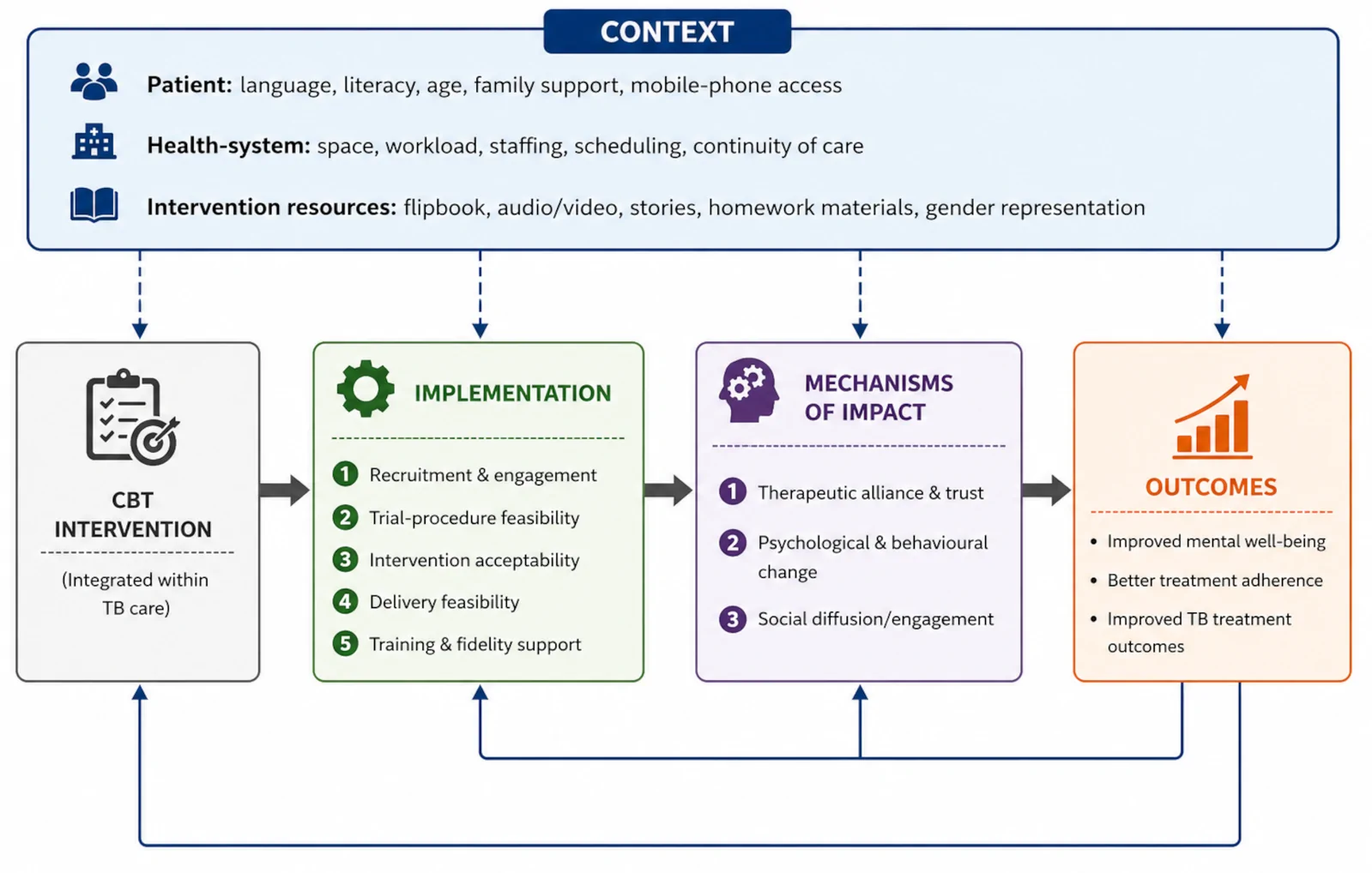
The conceptual framework of the process evaluation findings mapped under the MRC framework. CBT, cognitive behavioural therapy; MRC, Medical Research Council; TB, tuberculosis.

## Findings

### Implementation

#### Recruitment and trial procedures

A total of 80 participants were eligible and consented to participate in the in-built pilot during the 3-month recruitment period. One participant failed to complete the baseline assessment, leaving 79 participants who were randomly assigned, demonstrating efficient, timely enrolment in the two trial arms. The interview participants shared that regarding patient recruitment, the trial staff initially had difficulty convincing some patients’ families and explaining the project to them (*TS1*). They stated that DOTS facilitators (therapists) play a vital role as the first and most trusted point of contact for patients. They believe that if they provide information about the project, it will be easier for patients to understand (*TS2*). In interviews, patients also expressed satisfaction with the team’s assistance, which provided excellent guidance, clearly explained the intervention and offered support throughout their TB and mental health treatment (*Pt1*). They recalled being asked several questions during their initial visit for TB treatment, and they found those questions easy to answer. However, they could not remember all the details of the assessment questionnaires. One participant noted that the questions focused on their life circumstances and the challenges they faced due to the illness, but he did not remember the specific terms or questions (*Pt2*).

Among the interviewed participants, trial staff noted that when assessments took too long, patients would become irritated, which was a significant challenge in the field, especially because some patients were in pain due to TB symptoms and just wanted to take their medicine and leave quickly. Similarly, when repetitive questions related to mental health were asked, the patients would become uncomfortable and somewhat defensive. This was mostly related to the mental health stigma-related questions. However, the trial staff convincingly explained the purpose and importance of these assessments to the patients. Moreover, after the first session, the patients started to trust the team, and a bond developed between them (*TS3*).

#### Acceptability and feasibility of the CONTROL intervention

Across all participant groups (patients, therapists, MOs and trial staff), there was consistent support for the CONTROL programme’s acceptability and shared belief in its potential benefits for patients with TB. Patients were motivated to participate in this trial in the hope of a quick recovery and to have a supportive outlet for openly expressing their emotions.

The quantitative data demonstrated high retention in completing CBT sessions: 95% of participants completed at least four intervention sessions and 92% completed all sessions, indicating strong engagement and adherence to the therapy protocol. Interviewed patients also highlighted that participation in the sessions was feasible when the schedule was predetermined and communicated in advance (*Pt3*). They also mentioned that the CBT sessions lasted 30 min–1 hour, depending on each patient’s level of comprehension. Similarly, the follow-up completion was also substantial: 86% completed the 8-week follow-up, 81% the 24-week follow-up and 74% the 32-week follow-up. This was further supported by interview participants, who completed the entire series of sessions and agreed that six sessions were optimal for facilitating meaningful improvement in mental well-being. Patients reported satisfaction with the delivery of sessions but suggested that the session duration should be shortened (*Pt4*).

Therapists shared that in some facilities, a dedicated space was available for conducting CBT sessions, but in others, they had to adapt and use whichever room was available at the time (*TP1, TP2*). However, therapists expressed concern about the added workload and potential delays in patients’ routine TB care, particularly at sites with higher patient loads. They suggested allocating additional human resources to help manage patients alongside them (*TP3*).

Both therapists and patients reported that completing homework assignments was challenging for many patients, particularly those who were uneducated or had low literacy levels. As a result, patients often relied on therapists or family members for assistance, which at times made the process feel burdensome (*Pt5, TP4*). To improve homework participation, therapists emphasised the use of stickers on homework sheets. They also suggested redesigning the homework as a tick-box questionnaire that enables patients to indicate their responses by placing a cross, tick or sticker next to each item (*TP5*).

#### Fidelity of the intervention

Table 3 presents the item-wise and session-wise competency scores for therapists. The CTS-R domain scores ranged from 3.46 to 4.08, indicating that therapists consistently performed above the expected level of competence in all aspects of CBT delivery (exceeding the competency threshold of 3). The highest mean score was for Item 5 (M=4.08, SD=1.139), while the lowest was for Item 8 (M=3.46, SD=1.103). Among the six CBT sessions evaluated, Session 1 recorded the highest mean competency score (M=51.75, SD=16.81), while Session 3 had the lowest (M=39.75, SD=2.06) (table 4). Despite differences across sessions, all six session scores remained above the competency threshold of 36, indicating that therapists’ competence was adequate throughout.

**Table 3:** CBT training feedback from therapists participants

S. no	Training feedback		Frequency	Percentages
1	The training met my expectations	Strongly Agree	7	70.0
		Agree	3	30.0
2	Will be able to apply the knowledge in my role	Strongly agree	2	20.0
		Agree	8	80.0
3	The session was well-organised	Strongly agree	8	80.0
		Agree	2	20.0
4	The training activities and resources were useful and relevant	Strongly agree	5	50.0
		Agree	5	50.0
5	The trainer was knowledgeable	Strongly agree	9	90.0
		Agree	1	10.0
6	The quality of instruction was good	Strongly agree	6	60.0
		Agree	4	40.0
7	The trainer met the training objectives	Strongly agree	8	80.0
		Agree	2	20.0
8	Participation and interaction were encouraged	Strongly agree	8	80.0
		Agree	2	20.0
9	Adequate time was provided for questions and discussions	Strongly agree	5	50.0
		Agree	5	50.0

**Table 4:** Cognitive therapy scale-revised (CTS-R) scores: therapist competency across items and sessions

	Number of sessions (N)	Mean	Std. deviation
Item-wise competency level			
Item 1-Agenda setting and adherence	24	3.79	1.103
Item 2-Feedback	24	3.75	1.032
Item 3-Collaboration	24	4.00	1.180
Item 4-Pacing and efficient use of time	24	3.96	1.160
Item 5-Interpersonal effectiveness	24	4.08	1.139
Item 6-Eliciting of appropriate emotional expression	24	3.54	1.021
Item 7-Eliciting key cognitions	24	3.54	1.103
Item 8-Eliciting and planning behaviours	24	3.46	1.103
Item 9-Guided discovery	24	3.87	1.296
Item 10-Conceptual integration	24	3.67	0.963
Item 11-Application of change methods	24	3.75	1.032
Item 12-Homework setting	24	3.96	1.197
Session-wise competency level			
Session 1	4	51.75	16.81
Session 2	4	44.25	13.23
Session 3	4	39.75	2.06
Session 4	4	40.00	5.89
Session 5	4	49.25	13.38
Session 6	4	47.25	10.62

Figure 2 summarises the fidelity to content of the six CBT sessions delivered during the CONTROL pilot trial. For each session, the levels of content implementation were categorised either as ‘above partially implemented’ or ‘fully implemented.’ All four therapists (100%) implemented ‘Session 1’ at a level above partially implemented. For Session 2, three therapists (75%) achieved implementation above the partial level, while one therapist (25%) fully implemented the session. Session 3 demonstrated the highest fidelity, with all four therapists (100%) fully implementing the intervention. For Sessions 4 and 5, implementation was evenly divided: 50% of therapists delivered the session at a level above partially implemented, while the remaining 50% achieved full implementation. Session 6 was fully implemented by all therapists (100%). These findings suggest high levels of content implementation fidelity across sessions. These quantitative findings were further supported by interviews with trial staff, who reported the critical role of therapists in delivering the CBT intervention effectively. Along with this, staff also recommended periodic refresher training to ensure ongoing fidelity and skill reinforcement among therapists (*TS4*).

**Figure 2:**
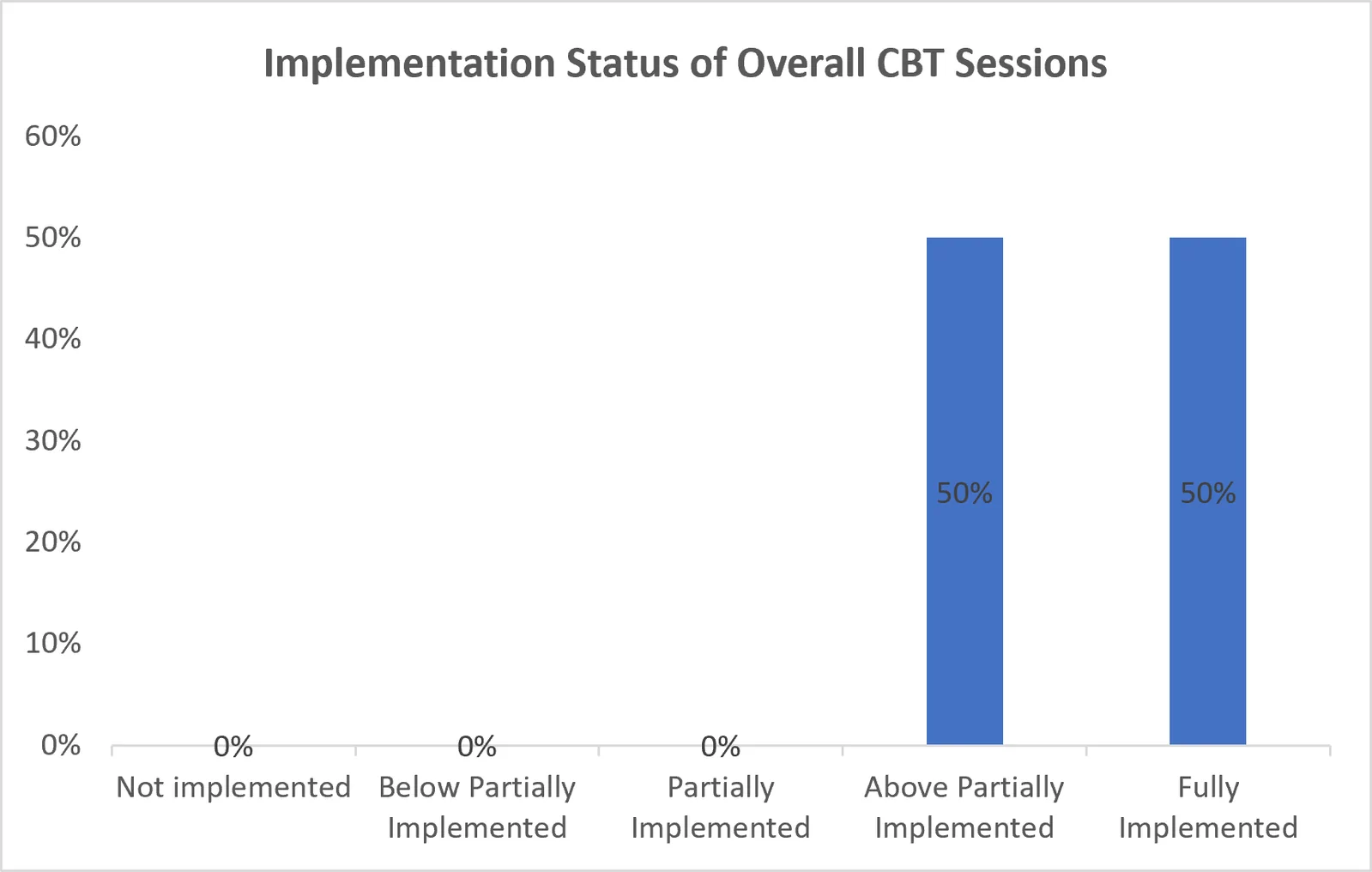
Implementation fidelity was high, with 50% of sessions rated as above partially implemented and 50% as fully implemented. CBT, cognitive behavioural therapy.

#### Training implementation

Two structured training sessions were held to enhance the skills of TBHWs in delivering the intervention. One of these was a 5 day training involving therapists (DOTS facilitators) from 10 TB facilities across Peshawar and Haripur. Attendance records confirmed full participation, with all 10 nominated participants completing the training within the scheduled time. Additionally, table 3 summarises participant feedback on the CBT training. Overall, responses indicate high satisfaction, with most participants endorsing ‘strongly agree’ across key indicators. Specifically, 90% of participants strongly agreed that the trainer was knowledgeable, and 80% reported that the sessions were well-organised and promoted active participation. All participants either agreed or strongly agreed that the training met their expectations and was relevant to their professional roles. Responses also indicated satisfaction with the instructional quality and the time allocated for discussion. The findings suggest that the training was generally well-received and considered relevant.

These quantitative findings were further supported by qualitative interviews, in which feedback on the training was consistently positive across therapists, MOs and trial staff. Therapists found the training beneficial for both enhancing patient care and their personal development, while MOs viewed it as a valuable supplement to their current duties (*TP6*). Most of the trial staff found the training to be helpful and effective in their fieldwork, and they believed that more practice made them better at performing. However, they emphasised that incorporating comprehensive theoretical knowledge into training should be complemented by a strong focus on preparing participants for practical challenges, highlighting the importance of aligning training with real-world field contexts (*TS 5*). Participants suggested running structured mock training sessions, where new assessors could first observe these simulations and interview demonstrations to improve their understanding before beginning fieldwork (*TS 6*).

### Mechanisms of impact

#### Therapeutic alliance and trust

Quantitative competency assessment demonstrated that therapists consistently scored high on the CTS-R domains of guided discovery (mean=3.87), collaboration (mean=4.00), feedback skills (mean=3.75) and interpersonal effectiveness (mean=4.08) (table 4). This indicates that they were able to establish therapeutic relationships while actively involving patients throughout CBT sessions, whereas domains of eliciting and planning behaviours (mean=3.46), eliciting emotional expression (mean=3.54) and eliciting key cognitions (mean=3.54) (table 4) represented areas requiring relatively greater skill development. These quantitative findings converged with the qualitative accounts from patients who reported that therapists were dedicated to their care and demonstrated an understanding of their illness. The bidirectional communication during CBT sessions facilitated the development of a therapeutic relationship, enabling patients to openly share their feelings and concerns (Pt 6, Pt 7).

Interviewed therapists also reported that more time with patients during CBT delivery strengthened the therapeutic relationship, and empathetic care often alleviated patients’ illnesses. MOs advocated for the continuation of the programme, emphasising its perceived benefits for patients (MO 1). Together, these quantitative and qualitative findings suggest that competent CBT delivery facilitated the development of therapeutic relationships, which may have strengthened participant engagement and intervention acceptability.

#### Psychological and behavioural changes

Quantitative competency assessment demonstrated satisfactory therapist performance in application of change methods (mean=3.75) and homework setting (mean=3.96) (table 4), suggesting that cognitive and behavioural techniques were delivered as intended. This aligned with patients’ accounts that CBT sessions were understandable, relevant and contributed to positive psychological and behavioural changes (*Pt 8*). These mental health improvements led to increased motivation across multiple domains of day-to-day life, including resuming spiritual practices, pursuing vocational interests and adhering to treatment. Moreover, many patients reported improved communication and stronger relationships with their families because of these sessions. The positive impact of CBT was further supported by therapists’ feedback, who confirmed that patients receiving CBT sessions showed increased emotional stability, even during the challenging course of TB treatment (*TP 7*).

### Context of the CONTROL intervention

#### Patients’ communication and engagement

The patients’ capacity to process information about the trial procedures and assessment was mainly influenced by factors such as language, educational background and age. Patients, therapists and trial staff emphasised that communicating in a language familiar to the patient was crucial in cultivating trust and rapport, ensuring that patients fully understood the discussions (*TP8*). Therapists and trial staff observed that illiterate patients often struggled to grasp information quickly. Although detailed explanations helped, it required a significant amount of time. Therapists demonstrated satisfactory competency in pacing and efficient use of time (mean=3.96) (table 4), although in qualitative interviews trial staff and therapists shared that older patients exhibited reduced comprehension, appearing passive and unresponsive, necessitating repeated explanations to facilitate better understanding (*TS 7*).

Participants (therapists and patients) explained that the CBT session content, particularly the flipbook, was instrumental in enhancing both understanding of the treatment and recall of the information. The flipbook’s visual approach to content delivery facilitated patient comprehension, especially among uneducated patients (*Pt 9, TP9*). Patients reported that the CBT content was helpful, noting that relatable therapist-narrated stories and session audio provided meaningful support for their recovery. Therapists adapted storytelling to enhance recall; however, limited patient access to mobile phones hindered the effective use of audio materials (*TP10*).

#### Intervention resources

Although participants viewed the intervention materials positively, they identified several opportunities to strengthen intervention delivery. They stated that ‘session six’ of the flipbook lacked a female character and recommended adding a female-led story to improve gender representation (*TP11*). The associated video content was considered useful but needed more detail for better comprehension. They also proposed providing financial support for patients without mobile phones to help them access audio materials. In addition, they suggested a 2-week interval between sessions to improve workload management and advised synchronising CBT sessions with routine patient visits for TB medication refills (*TP12*). One of the patients also suggested promoting awareness of this mental health treatment among the community through social media platforms such as TikTok and SMS to encourage greater engagement.

MOs who delivered mhGAP as ETAU provided suggestions, including implementing structured tools such as follow-up cards or mhGAP stamps on patient records to help track mental health treatment progress and schedule follow-up sessions (*MO2*). They further highlighted the need to establish a formal system for the seamless transfer of patients between treatment centres when continuing care at the original site is not feasible, thereby ensuring treatment continuity.

## Discussion

This paper presents critical insights from the pilot phase of the CONTROL trial on the acceptability, feasibility and fidelity of a CBT-based intervention, demonstrating its practical application and providing evidence to refine its design and delivery for greater impact before the definitive trial. In line with the MRC framework used to structure the results, the discussion below is organised around the three core process evaluation domains: implementation, mechanisms of impact and context and its implications for the definitive CONTROL trial. Logistical challenges, such as space constraints and difficulties completing homework among low-literacy patients, indicate areas that need adjustment. Contextual strategies, including language adaptation, flexible scheduling and the use of visual and audio materials, proved essential for maintaining engagement. These findings confirm the intervention’s promise while highlighting the need to refine delivery methods for improved scalability and integration within the national TB programme.

The literature highlights that process evaluation is significant in public health research because it helps clarify the mechanisms and contextual factors associated with outcome differences, identifies barriers and facilitators to intervention delivery and guides more accurate intervention adaptation.^35 36^ Several studies have found that TB treatment interventions that provide enhanced patient contact or support are both well accepted by patients and associated with improved outcomes. Iribarren *et al*^37^ reported that a combined mobile app, drug metabolite test and treatment supporter intervention (TB-TSTs) had high acceptability and a treatment success rate of 95%, compared with 81% for usual TB care.^37^ Moreover, a systematic review showed that performance is better with more supportive and supervised treatment, such as DOTS, that provides additional patient education, counselling and medication reminders, compared with unsupervised or self-administered approaches.^38^ Similarly, our findings revealed that the CBT intervention was acceptable to participants. Moreover, the DOTS facilitators (referred to as the therapist in the study) spent more time with patients, thereby strengthening their therapeutic relationship and improving treatment outcomes. The high acceptability rate is further supported by the retention rate: 92% of participants completed all six CBT sessions, demonstrating strong engagement and commitment to the therapy plan.

Session scheduling remained flexible in this study, with days chosen based on the availability of both patients and therapists. In some instances, session durations were extended beyond 30 min for patients with lower educational backgrounds, depending on their understanding and dedicated spaces were organised for delivering sessions to ensure patient confidentiality. Likewise, research examining CBT delivery in constrained or real-world settings shows that flexibility in scheduling and session count enhances feasibility.^39^ Joseph Mignogna *et al* (2018) adapted brief CBT for primary care by shortening session lengths (35–40 vs ~45–50 min) and allowing modules to be split across multiple sessions when needed to accommodate both provider and patient schedules.^39^ Similarly, Alatiq and l Modayfer (2019) found that although CBT protocols typically recommend 12–16 sessions, their transdiagnostic protocol was flexible on session number, with most patients requiring far fewer; an average of five sessions overall and nine among those who completed treatment, indicating that session count could be tailored to individual need rather than fixed in advance.^40^

Fidelity was assessed in 10% of all CBT sessions; this sampling approach is consistent with previous fidelity studies^29 30^ and provided a practical means of evaluating intervention delivery within the resource constraints of the in-built pilot. Therapists consistently achieved CTS-R scores above the established competency threshold, indicating competence in delivering the intervention with fidelity to the CBT model. In the context of task-shifting, these findings are particularly important, as they demonstrate that trained non-specialist TBHWs were able to deliver a structured psychological intervention with adequate competence following structured training and supervision. Fidelity assessment is a key component of process evaluation because it helps determine whether intervention outcomes can reasonably be attributed to the intervention itself rather than to variations in its implementation.^26^ The consistently satisfactory competency scores therefore strengthen confidence that the training and supervision model was sufficient to support high-quality intervention delivery within routine TB services. These findings are consistent with evidence from LMICs showing that, with appropriate training and ongoing supervision, non-specialist health workers can competently deliver evidence-based psychological interventions, supporting the feasibility of task-shifted mental healthcare.^41^ In our study, the therapists and other staff members were satisfied (90% with the trainer’s knowledge) with their training; however, to further improve their skills and performance, they requested refresher courses and practical role-playing exercises. Consistent with prior literature, training approaches that include role-plays or behavioural rehearsal have been shown to improve therapist adherence, competence and skill retention, more so than lecture-based training alone.^42 43^

Although therapists showed high competency in guided discovery, feedback and applying change methods, this addresses whether the intervention was delivered as intended, not why it appeared to work. Patients described therapists as dedicated and able to build trust despite initial hesitancy, pointing to the therapeutic alliance as a plausible mechanism underlying engagement and perceived benefit. This is consistent with psychotherapy literature identifying alliance quality as one of the most robust predictors of outcome across therapeutic modalities.^44^ The comparatively lower scores for eliciting and planning behaviours and emotional expression suggest alliance-building skills may develop ahead of CBT’s more technical elements among newly trained non-specialist providers, an area the refresher training and role-play recommended above could target. Patients also reported psychological and behavioural improvements, including motivation to resume spiritual and vocational activities, treatment adherence and better family communication, attributed to the CBT sessions. These are clinically relevant beyond their intrinsic value: depression and psychological distress are established, independent risk factors for poor TB treatment adherence, loss to follow-up and mortality.^9^ This offers a plausible pathway from alliance-based CBT to the broader TB treatment benefits noted by staff and a testable mechanism for the definitive trial to examine alongside the process measures reported here.

Language, educational background and age influenced patients’ capacity to engage with trial procedures and CBT content. Communicating in a language familiar to the patient was consistently identified as central to building trust and understanding, while older and less literate patients required repeated, simplified explanations. To support comprehension across this range of literacy and language backgrounds, the intervention combined audio and video recordings, pictorial flipbooks and structured homework tasks to reinforce recall and practice between sessions. These practical, multimodal design features corresponded with higher levels of adherence to intervention content. Literature emphasises the importance of structured homework assignments for patient recall and practice^45^ and using imagery-based techniques alongside verbal CBT has similarly been shown to improve treatment outcomes.^46^ At the same time, participants identified concrete opportunities to strengthen these materials and their contextual fit, for example, adding a female-led story to the flipbook to improve gender representation, providing financial support for patients without mobile phones to enable audio-material access and better aligning session scheduling with routine TB medication visits. MOs similarly proposed structured tracking tools, such as follow-up cards or mhGAP stamps on patient records and a formal system for transferring patients between treatment centres to preserve continuity of mental healthcare. Taken together, these context-level findings suggest that the acceptability and apparent benefit of the CBT intervention depended not only on its clinical content but also on how well its delivery was adapted to patients’ literacy, language, resources and the existing TB care pathway; an interpretation consistent with implementation research from other task-shifted psychological interventions in LMICs, where similar contextual constraints necessitate flexible adaptation while maintaining fidelity to core treatment principles.^47^ Because this was an internal pilot ahead of a definitive trial, these process evaluation findings should be interpreted as evidence of feasibility and acceptability rather than clinical effectiveness,^48^ a distinction the definitive CONTROL trial is now positioned to address.

This study has several methodological limitations. The qualitative sample was limited to patients who attended at least four CBT sessions; those who discontinued earlier were not interviewed, which may have under-represented less favourable experiences of the intervention. As a process evaluation within a pilot RCT, it was not designed to examine cost-effectiveness, so the economic implications of the intervention remain unassessed. Fidelity was assessed through direct structured observation rather than audio or video recording, which may have missed some real-time protocol deviations; the presence of an observer may also have introduced a Hawthorne effect, with therapists adhering more closely to the protocol than they would in routine, unobserved practice. The definitive CONTROL trial will address these limitations through audio-recorded fidelity assessment, broader qualitative sampling irrespective of session attendance and a planned cost-effectiveness analysis.

## Conclusion

This process evaluation provides valuable insights into the feasibility, acceptability and contextual implementation of a task-shifted CBT intervention for depression among patients with TB within routine care settings in Pakistan. Findings indicate strong acceptability of the intervention among patients, DOTS facilitators, MOs and trial staff, underpinned by a positive therapeutic alliance and competent, fidelity-consistent delivery by trained non-specialist health workers. Participants reported improvements in mental well-being, motivation to adhere to treatment and better communication with healthcare providers. Several implementation challenges emerged, including limited mobile phone access, literacy barriers, space constraints and scheduling issues, prompting stakeholders to recommend revisions to intervention content, training and follow-up systems. As a pilot process evaluation, these findings speak to what was feasible and acceptable to deliver, the implementation knowledge, rather than to clinical effectiveness and should be interpreted with this scope in mind. They will directly inform refinements to the CBT intervention and trial procedures for the forthcoming definitive CONTROL trial, ensuring better contextual adaptation, improved implementation fidelity and greater integration within existing TB service frameworks. Future research should evaluate the intervention’s clinical effectiveness, scalability and cost-effectiveness to support broader policy and programme adoption.

## Supplementary material

10.1136/bmjopen-2025-115224 online supplemental table 1

10.1136/bmjopen-2025-115224 online supplemental file 1

10.1136/bmjopen-2025-115224 online supplemental file 2

## Data Availability

Data are available upon reasonable request.
