# Supplementary material for: Embedding cognitive behavioural therapy for depression among patients with tuberculosis in Khyber Pakhtunkhwa, Pakistan: insights from a pilot trial process evaluation
Source: BMJ Open. 2026 Nov 9;16(11):e115224. doi: 10.1136/bmjopen-2025-115224 (PMC13756412; doi:10.1136/bmjopen-2025-115224)
Supplement: Supplementary file 1 [file bmjopen-2025-115224.reviewer_comments.pdf]

# PEER REVIEW HISTORY

BMJ Open publishes all reviews undertaken for accepted manuscripts. Reviewers are asked to complete a checklist review form and are provided with free text boxes to elaborate on their assessment. These free text comments are reproduced below.

## ARTICLE DETAILS

### Title (Provisional)

Embedding cognitive behavioral therapy for depression among tuberculosis patients in Khyber Pakhtunkhwa, Pakistan: Insights from a Pilot Trial Process Evaluation

### Authors

Ahmad, Fayaz; Niaz, Aiman; Qazi, Fatima Khalid; Rasool, Shaista; Jawed, Rubab; Ullah, Asad; Fonseka, Nishani; Shiekh, Saima; khan, Zohaib; Farooq, Saeed

---

## VERSION 1 - REVIEW

---

| | |
|---|---|
| **Reviewer** | **1** |
| **Name** | **Al-Aghbari, Nuha** |
| **Affiliation** | **Sana'a University, Community Medicine Department** |
| **Date** | **24-Jan-2026** |
| **COI** | **None** |

---

While the manuscript is valuable in filling a critical information gap regarding integration of TB and mental health, there is a need to revise the paper. This is with the aim of strengthening the conceptual framework, qualitative component, and articulation of the work's significance to implementation science.

**Introducation**

- This study as contributing to implementation knowledge, not clinical effectiveness. Please revise this.
- I strongly recommend adding a short paragraph near the end of the Introduction that explain about: the implementation uncertainties existed before this pilot also what are the process for the evulation
- Your reference to the MRC framework is repeated but not analytical in any sense.
- You indicate that the evaluation focuses on the following Implementation, Mechanisms of Impact, Context, but in Results, themes are mostly in those experiental areas ("patients liked it" and so on), with little explicit reference to those areas.
- Your analysis lacks sufficient analytical signposting, I recommend:

✓ "Explicitly map each qualitative theme to:"
✓ Implementation (e.g., fidelity, dose, reach
✓ Mechanisms of Impact (e.g. Trust, Therapeutic Alliance, Stigma Reduction)
✓ Context (e.g. space constraints, literacy, family involvement).

**Method**

- May you justify why CBT sessions were chosen.
- Can you clarify if there a qualitative saturation was considered in this study?

Fidelity Assessment: Important, but under-theorised

You report fidelity and CTS-R scores clearly, but do not sufficiently interpret what "adequate fidelity" means in a task-shifting context.

- Is 10% observation enough?
- How may presence affect delivery?
- Which aspects of CBT proved more difficult than others to implement?

**Results**

- The tables are clear but little dense; ensure all are referenced explicitly in the text.
- Figure 1 would benefit from a short interpretive caption, not just description

**Disscusion**

It summarized the finding well but does not sufficiently engage with implementation literature, it need more sternghth.

---

| | |
|---|---|
| **Reviewer** | **2** |
| **Name** | **Sarkar, Sonali** |
| **Affiliation** | **JIPMER, Preventive and Social Medicine** |
| **Date** | **13-Jul-2026** |
| **COI** | **None** |

---

This is a good study on a topic of public health relevance. Depression is a common, but ignored problem among TB patients.

This manuscript reports the findings of process evaluation of a large trial, CONTROL (COgNitive Therapy for depRessiOn in TuberculLosis treatment) for improving depression in MDR-TB patients. appropriate frameworks have been used to report the findings.

Overall, the article is written well with adequate justification. However, it is lengthy and can be made more succinct. A few queries or suggestions are as follows.

1. Even though the CONTROL trial is for MDR-TB patients, it's not clear why the pilot trial was performed in DSTB patients.

2. Methods:

a) The rationale for conducting the number of SSIs and FGDs is not provided.

b) Justification for the timings of the interviews need to mentioned.

3. Results:

a) As the study used a mixed-methods design, the reporting of the results could have been integrated, such as a joint display.

b) Presentation of the qualitative results need to be improved. Under each theme, there can be many sub-themes and appropriate quotes can be provided for each of the corresponding sub-themes. Direction of the results need to be mentioned for these sub-themes under each of the theme.

c) Conceptual framework can be presented as a figure for better understanding of the qualitative results.

---

## VERSION 1 - AUTHOR RESPONSE

| **Reviewer 1** | | |
|---|---|---|
| 1. Introduction | | |
| a) | This study contributes to implementation knowledge, not clinical effectiveness. Please revise this. | We thank the reviewer for this valuable suggestion. We agree that this distinction could be made more explicit. Accordingly, we have revised the text in the Introduction (Lines 148–154) and the Conclusion (Lines 658–660) to more clearly emphasize the implementation focus of the study and ensure consistency throughout the manuscript. |
| b) | I strongly recommend adding a short paragraph near the end of the introduction that explains the implementation uncertainties that existed before this pilot, as well as the process for the evaluation. | We agree with the suggestion. We have added a paragraph near the end of the Introduction outlining the uncertainties that existed before the pilot. (Lines 164-172) This revision also improves the logical flow of the rationale and aims of the process evaluation. |
| c) | Your reference to the MRC framework is repeated but not analytical in any sense.<br>You indicate that the evaluation focuses on the following: Implementation, Mechanisms of Impact, Context, but in Results, themes are mostly in those experiential areas (“patients liked it” and so on), with little explicit reference to those areas.<br>Your analysis lacks sufficient analytical signposting. I recommend:<br>✓ "Explicitly map each qualitative theme to:"<br>✓ Implementation (e.g., fidelity, dose, reach<br>✓ Mechanisms of Impact (e.g., Trust, Therapeutic Alliance, Stigma Reduction)<br>✓ Context (e.g., space constraints, literacy, family involvement). | As per the suggestion, we have substantially revised the qualitative results to improve their analytical presentation. The findings are now explicitly organized according to the three domains of the MRC process evaluation framework: Implementation, Mechanisms of Impact, and Context, with clearly defined themes and subthemes. (Line 344-543) |

| 2. Methods | | |
|---|---|---|
| a) | May you justify why CBT sessions were chosen? | We thank the reviewer for this comment. As described in the published CONTROL trial protocol (Ref. 24), the pilot trial evaluated the CBT-based intervention developed as the core intervention of the CONTROL programme. Accordingly, this process evaluation focused on the implementation of the CBT intervention delivered during the pilot, as its primary purpose was to examine the acceptability, feasibility, and delivery of the intervention to inform refinement before the definitive trial. |
| b) | Can you clarify if there a qualitative saturation was considered in this study? | We thank the reviewer for this helpful comment. We have now clarified this in the Methods section. Specifically, we have added that data collection for the SSIs continued until thematic saturation was achieved, defined as the point at which no new codes or themes emerged from successive interviews. We have also clarified the rationale for the number of focus group discussions (FGDs), which was predetermined based on the predefined stakeholder groups included in the study (Line 200-209) |
| c) | Fidelity Assessment: Important, but under-theorised<br>You report fidelity and CTS-R scores clearly, but do not sufficiently interpret what<br>"Adequate fidelity" means in a task-shifting context. | Thank you for this observation. We have revised the Discussion to interpret our fidelity findings explicitly in relation to task-shifting: we now note that CTS-R scores exceeding the competency threshold are notable because they were achieved by non-specialist DOTS facilitators, and we link this to the wider task-shifting literature on non-specialist providers delivering psychological interventions competently with adequate training and supervision (lines 589-596). We have also added interpretation of comparatively lower sub-scores in specific competency domains, framing these as a target for refresher training ahead of the definitive trial (lines 596–601). |
| | Is 10% observation enough? | We selected **10%** of the intervention sessions for fidelity assessment in accordance with previous fidelity assessment studies, which have adopted a similar sampling proportion. The relevant references supporting this approach have been cited in the Methods section, (refs. 29- 30). In the pilot trial, CBT was delivered to 40 participants; therefore, assessing approximately 10% of the sessions provided a representative sample for evaluating fidelity while remaining feasible within the scope of the pilot process evaluation. We have also acknowledged this |

| | | |
|---|---|---|
| | | sampling approach in the Discussion section. (Line 586-588) |
| | How may presence affect delivery? | We thank the reviewer for this helpful comment. We acknowledge that the presence of an observer during fidelity assessment may have influenced therapists' behaviour, potentially leading them to adhere more closely to the CBT protocol than they might have under routine conditions (Hawthorne effect). This limitation has now been clarified in the Limitations section of the manuscript. |
| | Which aspects of CBT proved more difficult than others to implement? | This is addressed in the Results, where item-wise CTS-R competency scores (Table 4) show that although all domains exceeded the competency threshold, "eliciting and planning behaviours" (M = 3.46) and "eliciting emotional expression" (M = 3.54) were comparatively lower than domains such as guided discovery and pacing, indicating these more technical, action-oriented CBT components were relatively harder for non-specialist therapists to deliver (lines 468-474). Practical implementation challenges distinct from therapist competency, including homework completion among low-literacy patients, inconsistent availability of dedicated session space, and scheduling conflicts with routine TB care, are also reported (lines 406-407 and 411-412). The Discussion now interprets these competency differences explicitly, noting that alliance-building and relational skills may develop ahead of the more technical elements of CBT delivery among newly trained non-specialist providers, and identifies this as a specific target for refresher training in the definitive trial (lines 623-647). |
| 3. Results | | |
| a) | The tables are clear but a little dense; ensure all are referenced explicitly in the text. | We thank the reviewer for this comment. We have retained the tables in their current format, as the underlying detail (item-wise and session-wise competency scores, demographic breakdowns, training feedback distributions) is necessary for transparency and to support replication and future comparison across sites; we have therefore not simplified or split them further. We have checked and ensured that every table is now explicitly referenced at the corresponding point in the text. |
| b) | Figure 1 would benefit from a short interpretive caption, not just a description. | We have revised the caption of the previous Figure 1 (now Figure 2) to include a brief interpretive statement highlighting the high implementation fidelity observed across the evaluated CBT sessions. |

| | | |
|---|---|---|
| 4. Discussion | | |
| 1 | It summarizes the findings well but does not sufficiently engage with implementation literature; it needs more strength. | We thank the reviewer for this suggestion. We have revised and strengthened the Discussion by expanding the interpretation of our findings in relation to the implementation literature. |
| **Reviewer 2** | | |
| 1 | Even though the CONTROL trial is for MDR-TB patients, it's not clear why the pilot trial was performed in DSTB patients. | This appears to be a misunderstanding. CONTROL study is not MDR-TB focused. The CONTROL intervention was designed for both drug-susceptible tuberculosis (DS-TB) and multidrug-resistant tuberculosis (MDR-TB) patients. The pilot trial was therefore appropriately conducted among DS-TB patients as part of the intervention development and evaluation. We have revised the manuscript to clearly state that the CONTROL trial includes both DS-TB and MDR-TB populations (Line 138). |
| 2. Methods | | |
| a) | The rationale for conducting the number of SSIs and FGDs is not provided. | We have now clarified the rationale for the number of SSIs and FGDs in the Methods section. Specifically, we have added that the three FGDs were predetermined based on the predefined stakeholder groups, while the sample size for the SSIs was guided by the need to capture sufficient variation across stakeholder groups and continued until thematic saturation was achieved. (Line 202-209) |
| b) | Justification for the timings of the interviews needs to be mentioned. | We have now clarified the rationale for the timing and the duration of the qualitative interviews and focus group discussions on the Methods section. Specifically, we have added that the interviews were scheduled to align with key stages of the pilot trial, enabling participants to reflect on their experiences following intervention completion and follow-up assessments. (Line 221-225 and 261-263) |
| 3. Results | | |
| a) | As the study used a mixed-methods design, the reporting of the results could have been integrated, such as a joint display. | We have revised the Results section to strengthen the integration of the quantitative and qualitative findings using a narrative joint display approach. |
| b) | Presentation of the qualitative results need to be improved. Under each theme, there can be many sub-themes, and appropriate quotes can be provided for each of the corresponding sub-themes. Direction of the results need to be mentioned for these sub-themes under each of the theme. | The qualitative results have been reorganized into clearly defined themes and subthemes, with appropriate participant quotations presented in Table 3. |
| c) | Conceptual framework can be presented as a figure for better understanding of the qualitative results | We have added Figure 1 as a conceptual framework for the study, illustrating how the study findings are mapped onto the key |

| | | |
|---|---|---|
| | | domains of the MRC process evaluation framework. |

## VERSION 2 - REVIEW

| | |
|---|---|
| **Reviewer** | **2** |
| **Name** | **Sarkar, Sonali** |
| **Affiliation** | **JIPMER, Preventive and Social Medicine** |
| **Date** | **02-Sep-2026** |
| **COI** | |

Modification in the manuscript have adequately addressed the concerns of the reviewers except for the table 3, which remains the same. The numbered codes do not classify whether the participant was stating challenges, facilitators or describing the process that was followed in the pilot trial. Ideally under each sub-theme, codes are phrases signifying the import of the information obtained through qualitative interviews along with the direction, which clearly indicates the whether the experiences of the participants were favourable or unfavourable. These should be supported with few important quotes. Quotes are stated verbatim and it is good to mention some demographics and relevant information regarding experience of the participant if they were staff to make more sense of what they said.

## VERSION 2 - AUTHOR RESPONSE

| **Reviewer 2** | | |
|---|---|---|
| a) | Modification in the manuscript have adequately addressed the concerns of the reviewers except for the table 3, which remains the same. The numbered codes do not classify whether the participant was stating challenges, facilitators or describing the process that was followed in the pilot trial. Ideally under each sub-theme, codes are phrases signifying the import of the information obtained through qualitative interviews along with the direction, which clearly indicates the whether the experiences of the participants were favourable or unfavourable. These should be supported with few important quotes. Quotes are stated verbatim and it is good to mention some demographics and relevant information regarding experience of the | We thank the reviewer for this careful observation and apologise for the confusion caused by the previous version of the table. In that version, the column headed "Code" contained participant identifiers (e.g., TS1, Pt1) rather than qualitative codes. The adjacent "Participant" column listed only the participant category (patient, trial staff, therapist). We recognise that this labelling was misleading and did not convey the analytical meaning or direction of each excerpt.<br>Table 3 (now Supplementary Table S1) has been revised as follows:<br>Participant attribution: The mislabelled "Code" column has been removed. Each excerpt is now attributed with a single descriptor that provides the participant identifier, role, gender, data-collection method (IDI/FGD), age, and, for DOTS facilitators, years of professional experience. This gives context to each participant's account. |

| | | |
|---|---|---|
| | participant if they were staff to make more sense of what they said | Direction of experience: Each excerpt is now classified under its sub-theme as a Challenge (an unfavourable experience or barrier to implementation), a Facilitator (a favourable experience or enabler), or a Recommendation (an improvement proposed by the participant based on their experience).<br>Alignment with results: All excerpts were re-checked against the sub-themes and the results narrative.<br>The sub-themes were developed through [inductive/deductive/framework] thematic analysis. The full coding framework is available from the authors on request. |
| b) | If you have selected 'Yes' above, please provide details of any competing interests.: Not applicable | All the authors declare that there are no competing interests related to this study: Not applicable |
