# Supplementary material for: Embedding cognitive behavioural therapy for depression among patients with tuberculosis in Khyber Pakhtunkhwa, Pakistan: insights from a pilot trial process evaluation
Source: BMJ Open. 2026 Nov 9;16(11):e115224. doi: 10.1136/bmjopen-2025-115224 (PMC13756412; doi:10.1136/bmjopen-2025-115224)
Supplement: Supplementary file 2 [file bmjopen-16-11-s001.docx]

**Table S1: Representative themes with supporting participant quotes**

| **Subthemes** | **Challenges/**  **Facilitator/**  **Recommendation** | **Participant** | **Quote** |
| --- | --- | --- | --- |
| **Theme 1: Implementation** | | | |
| **Recruitment and trial procedures** | Facilitator | TS1-Trial Assessor-FGD- 27yrs-Female | We provided them with proper information and talked to them patiently, especially after their first session; their trust in us developed significantly. |
|  | Facilitator | TS2-Trial Coordinator-FGD-45yrs-Female | DOTS [therapist] is the first contact point of the patient, and if they give proper information about the study, then there is no problem in patient recruitment |
|  | Facilitator | Pt1-Patient-39yrs-Female-IDI | I was in a terrible condition. I was very worried and disappointed with my condition. Then I met a member of your team [pointed towards the Site Coordinator] who comforted and encouraged me, said my health would improve, and told me a little about this matter [means CBT therapy and research; she pointed towards the Flip book]. |
|  | Challenge | Pt2-Patient-57yrs-Male-IDI | Yes, they filled out some forms at that time after asking me, but I don't remember anything now because I was very unwell then. |
|  | Challenge | TS3-Trial-Psychologist-FGD-31yrs-Female | At times, when we asked about mental health directly, they felt uncomfortable and wondered why we were asking those questions. Patients would mind it, so we would find a way to ask the questions without them feeling uncomfortable. We would explain each question to them to help them understand. |
| **Acceptability and Feasibility of the CONTROL intervention** | Facilitator | Pt3-Patient-41yrs-Male-IDI | I would say that six sessions are the perfect amount of time. In this period, a person’s mind begins to improve, and they start to move away from hopelessness. According to the time they give, day by day, a person’s mental health improves, and the information they provide to the patient, the quicker the patient recovers. |
|  | Challenge | Pt4-Patient-32yrs-Male-IDI | I think the location and number of sessions are appropriate, but I would suggest shortening the CBT session length. |
|  | Facilitator | TP1-Therapist-DOT Facilitator-Male-FGD-5 Yrs of experience | Currently, it’s good. We have space. There is a separate room for CBT sessions and a separate room for the trial team. |
|  | Challenge | TP2-Therapist-DOT Facilitator- Male-FGD-22Yrs of experience | We don’t have any space at all. No separate space for CBT. Even the trial staff sit together in one room. |
|  | Challenge | TP3-Therapist-DOT Facilitator- Male-FGD-7 Yrs of experience | If CBT is integrated into the TB program, each patient will need to be screened, and we will have to provide CBT to as many patients as meet the criteria. This means that in the future, we will have 3 to 4 patients daily for CBT, which will be very difficult to manage. |
|  | Challenge | Pt5-Patient-63yrs-Female-IDI | No, when I came the next time, I told DOTs that no one could fill it [homework sheet] out for me. So, I then told them to fill it out for me. |
|  | Challenge | TP4-Therapist DOT Facilitator-Male-FGD-11Yrs of experience | The majority were uneducated, with only a few having studied up to matriculation or the intermediate level. So, when they brought back the homework, it was usually done by someone else. At times, the way we delivered the sessions differed from how they would complete the homework. |
|  | Recommendation | TP5-Therapist DOT Facilitator-Male-FGD-16 Yrs of experience | No, the stickers are very few and do not explain the detailed activities. The homework is quite detailed; they often write down their thoughts and share them, which cannot be done with stickers. If we turn the homework or activities into a questionnaire and give answers, I think it will be easier. Or we can put a sticker, tick mark, or cross in front of those questions. This will make it easier. |
| **Fidelity** | Recommendation | TS4-Trial Coordinator-45-Female-IDI | I suggest training for DOTS closer to the trial, as well as one or two refresher sessions, so that they can perform their jobs properly. |
| **Training implementation** | Facilitator | TP6-Therapist-DOT Facilitator-Male-FGD-8 Yrs of experience | It was excellent. The information provided during the training was substantial. When we applied that knowledge practically in the field, it was very beneficial. The best part was that the master trainers would visit us, and whenever we faced issues delivering the sessions, they would guide us. Overall, our experience was excellent. |
|  | Challenge | TS5-trial site coordinator-37yrs-FGD-Female | The training we received was very theoretical. We only understood this once we were in the field. The real understanding came from field experience. |
|  | Recommendation | TS6-Trial Assessor-FGD-29yrs -Male | Dummy training sessions should be conducted like the mock session we had before. New assessors should observe these sessions before going into the field. They should watch us conduct interviews to get a better understanding. |
|  | **Theme 2: Mechanism of Impact** | | |
| **Therapeutic alliance and trust** | Facilitator | Pt6-Patient-37yrs-Female-IDI | “When I first came here for treatment, I was very hopeless, and it's my habit that I don't trust anyone easily. He [therapist] worked very hard with me, and when he told me stories and showed me pictures from this flip book, my heart gradually became stronger. At that time, I thought that this person was working so hard with me and understood my illness so well.” |
|  | Facilitator | Pt7-Patient-57yrsFemale-IDI | “He [therapist] spoke very beautifully. If I struggled to understand something, he would repeat it for me until I understood.” |
|  | Facilitator | MO1-Medical Officer-Male-FGD-15 yrs experience | This program benefited both the patients and us. So, in my opinion, it should be continued. |
| **Psychological and behavioural changes** | Facilitator | Pt8-Patient-44yrs-Female-IDI | Yes, I've seen a lot of improvement since then. I used to cry frequently. I was worried about my health, and I didn’t know if I would ever get better. There was hopelessness. But slowly, I have started to feel better, and I no longer cry like that. |
|  | Facilitator | TP7-Therapist-DOT Facilitator-Male-FGD-15 Yrs of experience | Yes, in most cases, it [CBT intervention] met our expectations; I saw changes. For example, there was one educated patient who was very depressed. But after completing his sessions, and even while still undergoing TB treatment, he appeared very content and happy. He even told me that these sessions were very beneficial for him. I also noticed significant positive changes in many other patients. |
|  | **Theme 3: Context of the CONTROL intervention** | | |
| **Patients’ communication and engagement** | Facilitator | TP8-Therapist-DOT Facilitator-Male-FGD-10 Yrs of experience | Well, I feel that it wasn't a significant issue because if you establish communication with the patient, such as if they speak Pashto and we also speak Pashto, it creates a strong bond with the patient. Whatever needs to be said, we can easily explain to the patients, so it wasn't that problematic. |
|  | Challenge | TS7-Trial Psychologist-FGD-33yrs-Male | It led to detailed conversations with the patients. We delved deeper into our questions. Most patients were illiterate, so they wouldn't immediately understand the questions. We had to explain the issues to them in our own way, and then they would understand. |
|  | Facilitator | Pt9-Patient-66yrs-Male-IDI | Yes, in every session, they would show a picture from the book and then explain its details. |
|  | Facilitator | TP9-Therapist-DOT Facilitator-Male-FGD-18 Yrs of experience | We used the flipbook and handouts. We explained the stories written in them. Sometimes, I would create 1-2 stories myself based on the patient’s situation or environment. I also provided examples relevant to their conditions, using simple language so they could better understand the session. |
|  | Challenge | TP10-Therapist-DOT Facilitator-Male-FGD-13 Yrs of experience | Most patients didn't have mobile phones to listen to the audio files. So, we couldn’t provide audios to all patients. |
| **Intervention resources** | Recommendation | TP11-Therapist-DOT Facilitator- Male-FGD-8 Yrs of experience | Initially, we have Zarmina and Gul Muhammad's stories, but in the final session, only Gul Muhammad's story remains. When female patients come for the final [sixth] session, we start with Gul Muhammad's story, and they get confused. So, we must translate Gul Muhammad's story into Zarmina's. |
|  | Recommendation | TP12-Therapist-DOT Facilitator- Male-FGD-18 Yrs of experience | See, our main weapon is medicine, which we control. In the past, with the real DOT (Directly Observed Therapy), we used to call patients every week, and they would come happily. Therefore, if we provide 15 days of medicine instead of a month, we can also deliver CBT sessions in conjunction with that. |
|  | Recommendation | MO2-Medical Officer-Male-FGD-20 yrs experience | There should be a special card for follow-up visits after one month. That way, we can track who started treatment, since many patients come in on the same day. How would we know if they've started psychotherapy? |
