# Supplementary material for: Embedding cognitive behavioural therapy for depression among patients with tuberculosis in Khyber Pakhtunkhwa, Pakistan: insights from a pilot trial process evaluation
Source: BMJ Open. 2026 Nov 9;16(11):e115224. doi: 10.1136/bmjopen-2025-115224 (PMC13756412; doi:10.1136/bmjopen-2025-115224)
Supplement: Supplementary file 3 [file bmjopen-16-11-s002.docx]

**The CONTROL**

**(COgNitive Therapy for depRessiOn in tubercuLosis treatment) to improve outcomes for depression and TB in Pakistan and Afghanistan**

**Funded by: RIGHT3, NIHR**

**Reference: NIHR201773**

**CBT Delivery Assessment Scale**

**DOTs Name: _________________________________**

**Facility: _____________________________________**

Directions: Please carefully assess each item on a scale of 0 to 4 based on the following criteria.

***0= Not Implemented (0%)***

***1= Below Partially Implemented (10%-40%)***

***2= Partially Implemented (50%)***

***3= Above Partially Implemented (60%-90%)***

***4= Fully Implemented (90%-100%)***

| **Delivery of CBT content** | | **0** | **1** | | **3** | | **4** | | **5** | |
| --- | --- | --- | --- | --- | --- | --- | --- | --- | --- | --- |
| **Session 1: Psycho education and Behavioral Activation** | | | | | | |  | |  | |
| 1. Befriending | |  |  | |  | |  | |  | |
| 2. Agenda Setting objective 1: Psychoeducation about depression , anxiety & relationship of the session | |  |  | |  | |  | |  | |
| 3. Objective 2: Vicious cycle of depression & behavioral activation | |  |  | |  | |  | |  | |
| 4. Layout of the session | |  |  | |  | |  | |  | |
| 5. Information of basic concept of CBT (using elephant example | |  |  | |  | |  | |  | |
| 6. Process of therapy | |  |  | |  | |  | |  | |
| 7. Basic psychological symptoms of Depression | |  |  | |  | |  | |  | |
| 8. Basic physical symptoms of Depression | |  |  | |  | |  | |  | |
| 9. Causes of depression | |  |  | |  | |  | |  | |
| 10. Basic psychological symptoms of Anxiety | |  |  | |  | |  | |  | |
| 11. Basic physical symptoms of Anxiety | |  |  | |  | |  | |  | |
| 12. Relationship between TB & depression | |  |  | |  | |  | |  | |
| 13. Relationship between TB & anxiety | |  |  | |  | |  | |  | |
| 14. Treatment regarding anxiety and depression i.e., sleep hygiene | |  |  | |  | |  | |  | |
| 15. Other techniques: deep breathing exercise, thought distraction, guided imagery (these were thought to the lifestyle modification and healthy diet | |  |  | |  | |  | |  | |
| 16. Introducing Zarmina’s and Gul Muhammad’s characters | |  |  | |  | |  | |  | |
| 17. Behavior Activation: Information about vicious cycle. | |  |  | |  | |  | |  | |
| 18. Steps of behavioral activation | |  |  | |  | |  | |  | |
| 19. Homework assigned to the patient | |  |  | |  | |  | |  | |
| **Session 2: Motivational Interviewing** | | | | | | | | | | |
| 1. Befriending & Feedback of previous session (Homework check) | |  |  | |  | |  | |  | |
| 2. Agenda setting⁚ How to deal with TB, Medication adherence, Benefits and disadvantages of taking medicines, how confident you are of continuing treatment. | |  |  | |  | |  | |  | |
| 3. Objective 1: To resolve the confusion of patient and developing change plan | |  |  | |  | |  | |  | |
| 4. Objective 2: To make the patient confident and self-efficient that he/she has the ability to bring changes in their condition. | |  |  | |  | |  | |  | |
| 5. Brief introduction to Motivational Interviewing | |  |  | |  | |  | |  | |
| 6. Exploring confusion and importance | |  |  | |  | |  | |  | |
| 7. Assessing confidence and self-efficacy | |  |  | |  | |  | |  | |
| 8. Exploring personal goals and values | |  |  | |  | |  | |  | |
| 9. Developing a change plan through worksheet | |  |  | |  | |  | |  | |
| 10. Homework assigned to the patient | |  |  | |  | |  | |  | |
| 11. Agenda setting for the next session | |  |  | |  | |  | |  | |
| **Session 3: Cognitive reconstructing & Identifying thoughts** | | | | | | | | | | |
| 12. Befriending & Feedback of previous session (Homework check) |  | | |  | |  | |  | |  |
| 13. Agenda Setting   Objective 1 : Psychoeducation about Thought, emotions and physical symptoms & relationship of the session |  | | |  | |  | |  | |  |
| 14. Objective 2: Vicious cycle of Depression and anxiety |  | | |  | |  | |  | |  |
| 15. Layout of the session (21) |  | | |  | |  | |  | |  |
| 16. Basic concept of thought |  | | |  | |  | |  | |  |
| 17. Basic concept of emotions |  | | |  | |  | |  | |  |
| 18. Basic concept of behavior |  | | |  | |  | |  | |  |
| 19. Basic concept of Physical symptoms |  | | |  | |  | |  | |  |
| 20. Relationship between thought, emotion, behavior and physical symptoms |  | | |  | |  | |  | |  |
| 21. Relationship between TB & vicious cycle |  | | |  | |  | |  | |  |
| 22. Introducing Zarmina’s and Gul Muhammad’s diary no 1 |  | | |  | |  | |  | |  |
| 23. Homework assigned to the patient |  | | |  | |  | |  | |  |
| 24. Agenda setting for the next session |  | | |  | |  | |  | |  |
| **Session 4 : Cognitive restructuring & Challenging thoughts** | | | | | | | | | | |
| 1. Befriending & Feedback of previous session (Homework check) |  | | |  | |  | |  | |  |
| 2. Agenda Setting:   Objective 1: Psychoeducation about Cognitive restructuring |  | | |  | |  | |  | |  |
| 3. Objective 2: Cognitive distortions |  | | |  | |  | |  | |  |
| 4. Layout of the session |  | | |  | |  | |  | |  |
| 5. Basic concept of Cognitive distortions and cognitive restructuring |  | | |  | |  | |  | |  |
| 6. Introducing Zarmina’s and Gul Muhammad’s diary no 2 |  | | |  | |  | |  | |  |
| 7. Homework assigned to the patient |  | | |  | |  | |  | |  |
| **Session 5 : Cognitive restructuring & Alternative thoughts/balanced thoughts** | | | | | | | | | | |
| 1. Befriending & Feedback of previous session (Homework check) |  | | |  | |  | |  | |  |
| 2. Agenda Setting objective : Psychoeducation about Alternative thoughts |  | | |  | |  | |  | |  |
| 3. Layout of the session |  | | |  | |  | |  | |  |
| 4. Basic concept of Alternative thoughts |  | | |  | |  | |  | |  |
| 5. Introducing Zarmina’s and Gul Muhammad’s diary no 3 |  | | |  | |  | |  | |  |
| 6. Homework assigned to the patient |  | | |  | |  | |  | |  |
| **Session 6 : Problem Solving** | | | | | | | | | | |
| 1. Befriending & Feedback of previous session (Homework check) |  | | |  | |  | |  | |  |
| 2. Agenda Setting objective : Relationship btw depression & problem solving & problem-solving technique |  | | |  | |  | |  | |  |
| 3. Layout of the session |  | | |  | |  | |  | |  |
| 4. Process of five steps problem solving technique |  | | |  | |  | |  | |  |
| 5. Identifying problems |  | | |  | |  | |  | |  |
| 6. Brainstorm possible solution |  | | |  | |  | |  | |  |
| 7. Evaluate and select an alternative |  | | |  | |  | |  | |  |
| 8. Implement the chosen solution |  | | |  | |  | |  | |  |
| 9. Review the result |  | | |  | |  | |  | |  |
| 10. Introducing Gul Muhammad character with an example |  | | |  | |  | |  | |  |
| 11. Homework assigned to the patient |  | | |  | |  | |  | |  |
