# Supplementary material for: Embedding cognitive behavioural therapy for depression among patients with tuberculosis in Khyber Pakhtunkhwa, Pakistan: insights from a pilot trial process evaluation
Source: BMJ Open. 2026 Nov 9;16(11):e115224. doi: 10.1136/bmjopen-2025-115224 (PMC13756412; doi:10.1136/bmjopen-2025-115224)
Supplement: Supplementary file 4 [file bmjopen-16-11-s003.docx]

**Manual of the Revised Cognitive Therapy Scale (CTS-R)**

**Introduction**

This is a scale for measuring therapist competence in Cognitive Therapy and is based on the original Cognitive Therapy Scale. The CTS-R contains 12 items specified into two categories: General items and Cognitive therapy-specific items. The therapist should use specific tools i.e., items (1 to 5) should be used to conduct the session smoothly like sticking to a plan, giving feedback, working together with the patient, keeping a good pace, and being good at interacting with others. Whereas, items 6 to 12 are to guide and encourage the patients to discuss their specific problems.

These 12 items are,

| - **General items** | - **Cognitive therapy-specific items** |
| --- | --- |
| - Item 1: Agenda Setting & Adherence - Item 2: Feedback - Item 3: Collaboration - Item 4: Pacing and Efficient Use of Time - Item 5: Interpersonal Effectiveness . | - Item 1: Agenda Setting & Adherence - Item 6: Eliciting Appropriate Emotional Expression - Item 7: Eliciting Key Cognitions - Item 8: Eliciting Behaviors** - Item 9: Guided Discovery - Item 10: Conceptual Integration - Item 11: Application of Change Methods - Item 12: Homework Setting |

**Adapted Dreyfus Level of Competence**

The CTS-R includes the Dreyfus Model to evaluate how skilled a therapist is. The scale consists of six levels for assessment of therapist confidence which are,

- **Incompetent -** The therapist commits errors and displays poor and unacceptable behavior, leading to negative therapeutic consequences.
- **Novice -** At this level, the therapist displays a rigid adherence to taught rules and is unable to take account of situational factors. He/she is not yet showing any discretionary judgment.
- **Advanced Beginner** - The therapist treats all aspects of the task separately and gives equal importance to them. There is evidence of situational perspective and discretionary judgment.
- **Competent -** The therapist can see the tasks linked within a conceptual framework. He/she makes plans within this framework and uses standardized and routinized procedures.
- **Proficient -** The therapist sees the patient's problems holistically, prioritizes tasks and can make quick decisions. The therapist is skilled and able.
- **Expert -** The therapist no longer uses rules, guidelines, or maxims. He/she has a deep tacit understanding of the issues and can use novel problem-solving techniques. The skills are demonstrated even in the face of difficulties (e.g. excessive avoidance).

**Scoring system**

You will rate every item on a scale ranging from 0 to 6. You can assess the therapist's performance in many areas by referring to the detailed descriptions provided for each number. Simply use whole or half numbers to mark or circle the line that best expresses your feelings about the therapist's performance. Your decision over the rating to select will be aided by the descriptions on the right.

**Demographics:**

Please record the following demographics for each session you are going to observe.

| **District: _______________** | **Facility name: _______________** |
| --- | --- |
| **Patient ID:________________** | **Patient’s age: ________________** |
| **Patient’s Gender: ______________** | **Patient’s education: ______________** |
| **Therapist’s Designation: _____________** | **Therapist’s Gender: _____________** |
| **Session No: ___________________** | **Session starting time: ____________** |
| **Session end time: _____________** | **Total time taken: ____________** |

**ITEM 1 - AGENDA SETTING & ADHERENCE**

The agenda refers to the plan set between the therapist and the patient regarding the patient’s problem. The agenda for the therapy should be set appropriately. The conversation/therapy should focus on a specific target i.e., clear, relevant/specific, and realistic based on patients’ problems. This agenda may vary according to the stage of therapy.

**To assess this item/theme, first respond to the following questions,**

1. Did the therapist set an agenda with clear, discrete, and realistic goals - and adhere to it?

(A.	Excellent	B. Good	C. Fair	D. Poor)

2. Can you identify at least two specific agenda items?

(1. ___________	2. ______________)

3. Do you think the patient clearly understood what the therapy was going to cover?

(A.	Excellent	B. Good	C. Fair	D. Poor)

4. Did the agenda seem appropriate?

(A.	Excellent	B. Good	C. Fair	D. Poor)

5. Were the items prioritized?

(A.	Excellent	B. Good	C. Fair	D. Poor)

6. Was the session set sensitively?

(A.	Excellent	B. Good	C. Fair	D. Poor)

**Based on the above questions, consider these three features for scoring this item:**

(i) presence/absence of an agenda

(ii) Appropriateness of the contents of the agenda

(iii) Appropriate adherence to the agenda.

**Mark or Circle on the vertical line/Scale, the level to which you think the therapist has fulfilled the core function.**

**ITEM 2 – FEEDBACK**

In this item, Feedback refers to two-way communication that is communication between the patient and the therapist throughout the whole session. It also helps both the therapist and the patient to determine whether they have a mutual understanding of the problems and concerns.

Feedback ensures that patient understands the therapist's interventions, formulations and lines of their discussion. The major features of the feedback should focus on new material and highlight issues that require further clarification, according to patients' knowledge and attitudes. It also allows the individual to express positive and negative reactions regarding the therapy.

**To assess this item/theme, first respond to the following questions,**

1. Do you think the feedback was appropriate and sufficiently frequent?

(A.	Excellent	B. Good	C. Fair	D. Poor)

2. Up to what extent the therapist gets a piece of the information to provide a platform for new insight?

(A.	Excellent	B. Good	C. Fair	D. Poor)

3. Was the patient encouraged to provide feedback throughout the session?

(A.	Excellent	B. Good	C. Fair	D. Poor)

4. Do you think that the feedback was used effectively in helping the patient's understanding?

(A.	Excellent	B. Good	C. Fair	D. Poor)

**Based on the above questions, consider these three features for scoring this item:**

(i) Presence and frequency, or absence, of feedback. Feedback should be given/elicited throughout the therapy – with major summaries both at the beginning (review of the week) and end (session summary), while topic reviews (i.e. chunking) should occur throughout the session.

(ii) Appropriateness of the contents of the feedback.

(iii) Manner of its delivery and impression.

**Mark or Circle on the vertical line/Scale, the level to which you think the therapist has fulfilled the core function.**

**Item 3 – Collaboration**

The therapist should adopt a style that promotes a balanced relationship, whereby he/she and the patient work actively towards shared goals (e.g. through questioning techniques, shared problem-solving and decision-making). This is achieved by the development of a 'teamwork' approach. Hence the therapist should avoid being overly directive, too intellectual, controlling, or passive.

Good Collaboration will also involve a balance between verbal and non-verbal features. For example, deciding when to talk and when to listen; when to confront and when to back off; when to offer suggestions and when to wait for the patient to devise his/her own.

**To assess this item/theme, first respond to the following questions.**

1. Was the patient encouraged to participate fully as a team member?

(A. Yes	B. No	C. Somehow)

2. Was the therapist able to establish a collaborative relationship?

(A. Yes	B. No	C. Somehow)

3. Did the therapist give the patient sufficient space and time to think?

(A. Yes	B. No	C. Somehow)

4. Was the therapist overly directive or too controlling?

(A. Yes	B. No	C. Somehow)

**Based on the above questions, consider these three features for scoring this item:**

Three features need to be considered: the therapist's style should encourage effective teamwork through His/her use of:

(i) Verbal skills (e.g. non-hectoring/ non-aggressive)

(ii) Non-verbal skills (e.g. attention and use of joint activities)

(iii) Sharing of written summaries.

**Mark or Circle on the vertical line/Scale, the level to which you think the therapist has fulfilled the core function**

**ITEM 4 - PACING AND EFFICIENT USE OF TIME**

The session should be well time managed, such that it is neither too slow nor too quick. He/she must maintain sufficient control, limit discussion of peripheral issues, interrupt unproductive discussion, and pace the session appropriately. The pacing of the material should always be accommodated to the patient's needs and speed of learning. For example, when there is evidence of difficulties (e.g. emotional or cognitive difficulties), more time and attention may need to be given. In such circumstances, the agenda items may be shuffled or adapted accordingly. In some extreme circumstances (e.g. disclosure of suicidal thoughts), the structure and pacing of the session will need to change drastically following the needs of the situation.

**To assess this item/theme, first respond to the following questions**

1. Was the therapist able to recognize the patient's need and adapt the session accordingly?

(A. Yes	B. No	C. Somehow)

2. Was there any time during the session when the session moved too slowly/quickly (e.g. agenda setting phase)?

(A. Yes	B. No)

3. Do you think the session flowed well overall?

(A. Yes	B. No	C. Somehow)

4. Was the therapist able to avoid unproductive discussions?

(A. Yes	B. No	C. Somehow)

5. Was there sufficient time left for the homework assignment?

(A. Yes	B. No)

6. Was the pacing of the session adapted well to the needs of the patient?

(A. Yes	B. No	C. Somehow)

7. Did the patient appear rushed?

(A. Yes	B. No)

8. Dose the Therapist appear rushed?

(A. Yes	B. No)

**Based on the above questions, consider these three features for scoring this item:**

Three features need to be considered:

(i) The degree to which the session flows smoothly through the discrete phases;

(ii) The appropriateness of the pacing throughout the session;

(iii) The degree of fit to the learning speed of the patient.

**Mark or Circle on the vertical line/Scale, the level to which you think the therapist has fulfilled the core function**

**Item 5 - Interpersonal Effectiveness**

The ability of the therapist to form a good relationship with the patient is very important for good therapy. Indeed, for the patient to be able to disclose difficult material, there must be both trust and confidence in the therapist. The patient is put at ease by the therapist's verbal and non-verbal (e.g. listening skills) behavior. The patient should feel that the core conditions (i.e. warmth, genuineness, empathy and understanding) are present. However, it is important to keep professional boundaries.

**To assess this item/theme, first respond to the following questions.**

1. Did you consider the relationship was positive?

(A. Yes	B. No	C. Somehow)

2. Was the therapist displaying appropriate empathy, understanding, warmth and genuineness?

(A. Yes	B. No	C. Somehow)

3. Did therapist appear appropriately genuine, helping to facilitate therapeutic trust?

(A. Yes	B. No	C. Somehow)

4. Do you think therapist showed acceptance and liking (acceptance is fine) of the individual while remaining within Professional boundaries?

(A. Yes	B. No	C. Somehow)

5. Did the therapist appear confident?

(A. Yes	B. No	C. Somehow)

6. Did the therapist empathize with the patient’s distress?

(A. Yes	B. No	C. Somehow)

7. Did the therapist acknowledge any difficulties?

(A. Yes	B. No	C. Somehow)

**Based on the above questions, consider these three features for scoring this item:**

(i) Empathy - the therapist can understand and enter the patient's feelings imaginatively and uses this understanding to promote change;

(ii) Genuineness - the therapist has established a trusting working relationship;

(iii) Warmth - the patient seems to feel liked and accepted by the therapist.

**Mark or Circle on the vertical line/Scale, the level to which you think the therapist has fulfilled the core function**

**ITEM 6 — ELICITING OF APPROPRIATE EMOTIONAL EXPRESSION**

A therapist needs to address and handle the emotions that emerge during therapy sessions. They should be able to use their words and body language to either increase or decrease their emotional surroundings. The therapist should then be proficient in controlling the patient's emotions to assist them in making positive changes (e.g. hostility, anxiety, excessive anger). Effective facilitation will enable the patient to access and express his/her emotions in a way that facilitates change. Asking relevant questions may help a patient uncover the emotions that are connected to an unpleasant experience, for example, if they are not expressing any distressing feelings when discussing it.

**Respond to the following questions after observing the therapy session to address the emotional expression of both the therapist and patients during the session.**

1. To what extent the therapist paid sufficient attention to the person's emotions?
(A. Excellent	B. Good	C. Fair	D. Poor)
2. Did the therapist help the patient to differentiate between different emotions?
(A. Excellent	B. Good	C. Fair	D. Poor)
3. How effectively the therapist sensitively raise emotional topics?
(A. Excellent	B. Good	C. Fair	D. Poor)
4. Did the emotional intensity reach an ideal level to encourage positive change?
(A. Excellent	B. Good	C. Fair	D. Poor)
5. How well did the therapist's activity serve to motivate the patient appropriately?
(A. Excellent	B. Good	C. Fair	D. Poor)
6. Did the therapist prepare the patient to work on his/her emotions?
(A. Excellent	B. Good	C. Fair	D. Poor)
7. How effectively did the therapist manage emotional outbursts/scenes?
(A. Excellent	B. Good	C. Fair	D. Poor)

**Three features have to be considered:**

(i) Facilitation of access to a range of emotions;

(ii) Appropriate use and containment of emotional expression;

(iii) Facilitation of emotional expression; encouraging appropriate access and differentiation of emotions.

**Mark or Circle on the vertical line/Scale, the level to which you think the therapist has fulfilled the core function.**

**Item 7 - Eliciting Key Cognitions**

It is important to note that therapists frequently use various strategies to identify key concepts, such as thought diaries or downward arrow (technique name) approaches (uncovering essential core beliefs and belief systems that influence the patients’ thoughts, behaviors, and moods). Instead of falling under the heading of "applying methods for change," these approaches should be assessed in the context of this discussion. This area focuses mainly on methods for effectively changing attitudes and actions. A therapist should be able to recognize and extract the key cognitions, ideas, images, and beliefs that are important to the patient's distress. Key cognitions are frequently negative habitual self-statements or ideas about one's being and surroundings that generate or prolong unpleasant emotions.

**Share your feedback on the therapist's ability to draw significant ideas and cognitive patterns during the therapy session. Respond to the questions based on your today’s observation**.

1. Was the therapist able to identify and elicit the appropriate cognitions and biases?

(A. Yes	B. No	C. Somehow)

2. Was the therapist able to access and work with key cognitions?

(A. Yes	B. No	C. Somehow)

3. Was the therapist able to identify thinking biases and elicit hot cognitions?

(A. Yes	B. No	C. Somehow)

4. Were the cognitions elicited well?

(A. Yes	B. No	C. Somehow)

5. Does the therapist adequately demonstrate to the patient how to identify key cognitions

and biases?

(A. Yes	B. No	C. Somehow)

**Based on the above questions, consider these three features for scoring this item:**

(i) Eliciting cognitions that are associated with distressing emotions (i.e. selecting key cognitions or hot thoughts).

(ii) The skillfulness and breadth of the methods used (i.e. Socratic questioning; appropriate monitoring, downward arrowing, imagery, role-plays, etc.).

(iii) Choosing the appropriate level of work for the stage of therapy (i.e. automatic thoughts, assumptions, or core beliefs).

**Mark or Circle on the vertical line/Scale, the level to which you think the therapist has fulfilled the core function.**

**ITEM 8 - ELICITING AND PLANNING BEHAVIOURS**

Behavioral problems are observed frequently in psychiatric disorders. They take numerous forms, including withdrawal, avoidance, compulsions and various types of safety-seeking behaviors.

It is important to examine the role that behaviors were playing in triggering and maintaining the patient’s disorder. Behaviors often reinforce both negative thoughts and feelings. For example, the typical avoidance observed in social phobia prevents the person overcoming his/her fear, and obtaining the skills necessary to engage in social interactions. Some activities can be termed “safety-seeking behaviors” as patients employ them as a means of reducing their levels of distress (e.g. self-monitoring procedures, holding on tightly to objects).

The overall aim of this item is to help the patient gain insight into the effect of his/her behaviors concerning the problems. This can be done through the use of questioning, diaries and monitoring procedures.

**Respond to the following questions after observing the therapy session to address whether the therapist digs out the safety behaviors of the Patient during the session.**

1. Did the therapist examine adequately the role that behavioral features played in the triggering and maintenance of the patient’s problems?

(A. Yes	B. No	C. Somehow)

2. Did the therapist help the patient discover the impact of his/her behaviors in terms of relevant emotional features?

(A. Yes	B. No	C. Somehow)

**Based on the above questions, consider these two features for scoring this item:**

(i) Eliciting behaviors that are associated with distressing emotions (including, the use of safety-seeking Behaviors).

(ii) The skillfulness and breadth of the methods used (i.e. Socratic questioning; appropriate Monitoring, imagery, role-plays, etc.).

**Mark or Circle on the vertical line/Scale, the level to which you think the therapist has fulfilled the core function.**

**ITEM 9 - GUIDED DISCOVERY**

Guided discovery is a form of questioning that helps the patient to gain new perspectives for himself/herself without any debate or lecturing. The therapist should use the “questioning technique” to inquire about Patients problems. These questions must be based on the principle of the Socratic dialogues. With this approach, the patient receives guidance in formulating ideas about their current situation and generates ideas for potential solutions on their own. Effective guided discovery allows the therapist to express doubt on the patient's judgments in areas where they were previously sure, allowing for fresh insights and revision.

We have two categories for the questions:

- Good questioning style: Good questions are those asked in the spirit of inquiry.
- Bad questioning style: Bad ones are those that lead the patient to a predetermined/scheduled conclusion.

**Respond to the following questions after observing the therapy session to address that the guided discovery happened between the therapist and the patient during the session.**

1. Has the therapist used appropriate questions?

(A. Yes	B. No	C. Somehow)

2. Were the questions asked to facilitate the patient's understanding?

(A. Yes	B. No	C. Somehow)

3. Did the questions lead to or promote change?

(A. Yes	B. No	C. Somehow)

**Based on the above questions, consider these two features for scoring this item:**

(i) The style of the therapist - this should be open and inquisitive

(ii) The effective use of questioning techniques (e.g. Socratic questions) should encourage the patient to discover useful information that can be used to help him/her gain a better level of understanding.

**Mark or Circle on the vertical line/Scale, the level to which you think the therapist has fulfilled the core function.**

**ITEM 10 - CONCEPTUAL INTEGRATION**

In therapy, conceptualization is a knowledge-based process that enhances understanding and makes positive change possible. It aids the patient in understanding the cognitive meaning of their illness. Additionally, it enables the patient to understand the coping mechanisms and previous thoughts that were used to tackle the problems. The patient is introduced to the therapy understanding (i.e., the connections between "thoughts → feelings → behaviors") as part of the conceptualization process. The therapist's role is to support the patient in learning how his or her condition is associated with perceptions and interpretations, beliefs, attitudes, and expectations. A good conceptualization will examine previous cognitions and coping strategies as well as current ones. This theory-based understanding should be well integrated and used to guide the therapy forward.

**Respond to the following questions after observing the therapy session.**

1. Has the therapist socialized the patient to the CT rationale?

(A. Yes	B. No	C. Somehow)

2. Does the therapist demonstrate a good understanding of the Intervention developed CT?

(A. Yes	B. No	C. Somehow)

3. Does the patient have an adequate CT understanding of the problem?

(A. Yes	B. No	C. Somehow)

4. If you asked the patient about his/her problems, would he/she be able to explain it according to the CT model.

(A. Yes	B. No	C. Somehow)

6. Has the conceptualization been truly integrated (i.e. has it been used to guide the therapy)

(A. Yes	B. No	C. Somehow)

**Based on the above questions, consider these two features for scoring this item:**

(i) The presence/absence of an appropriate conceptualization that is in line with the goals of therapy;

(ii) How the conceptualization is used (e.g. used as the platform for interventions, homework, etc.).

**Mark or Circle on the vertical line/Scale, the level to which you think the therapist has fulfilled the core function.**

**Item 11 - Application of Change Methods**

To facilitate therapeutic change, therapists use change techniques, which are behavioral and cognitive strategies that correlate with the cognitive process and/or formulation. The effectiveness of change techniques depends on how well they are executed and whether or not they are used at the right stage in therapy.

In compliance with the formulation of this technique, the therapist skillfully uses and supports the patient in using suitable cognitive and behavioral strategies. The therapist also helps the patient to develop an appropriate cognitive method to evaluate the key cognitions associated with distressing emotions, leading to major new perspectives and shifts in emotions. The methods also allow the therapist to obtain feedback regarding the patient's level of understanding of prospective practical assignments (i.e. by the patient performing the task in-session).

**Respond to the following questions after observing the therapy session.**

1. Has the therapist ensured that the patient understands the rationale underpinning the method of the session?

(A. Yes	B. No	C. Somehow)

2. Was the method conducted skillfully?

(A. Yes	B. No	C. Somehow)

2. Were the learning goals achieved?

(A. Yes	B. No)

3. Was the technique consistent with the Intervention developed?

(A. Yes	B. No	C. Somehow)

4. Were the techniques administered with skill?

(A. Yes	B. No	C. Somehow)

5. Before using the techniques were the learning goals established?

(A. Yes	B. No	C. Somehow)

6. Where necessary, was a competent explanation of the rationale of the technique given?

(A. Yes	B. No	C. Somehow)

**Based on this checklist, now encircle the scale the level to which you think the therapist has fulfilled the core function of item-11 i.e., the Change technique. Keep in mind these three aspects of this item:**

(i) The appropriateness and range of both cognitive methods (e.g. Cognitive change diaries, continua, distancing, responsibility charts, evaluating alternatives, examining pros and cons, determining meanings, imagery restructuring, etc.) And behavioral methods (e.g. Behavioral diaries, behavioral tests, role play, graded task assignments, response prevention, reinforcement of patient's work, modeling, applied relaxation, controlled breathing, etc.);

(ii) The skill in the application of the methods - however, skills such as feedback, interpersonal effectiveness, etc. Should be rated separately under their appropriate items;

(iii) The suitability of the methods for the needs of the patient (i.e. neither too difficult nor complex).

**Mark or Circle on the vertical line/Scale, the level to which you think the therapist has fulfilled the core function.**

**Item 12 - Homework Setting**

Therapy must succeed when patients can apply the knowledge they gain in sessions to their daily lives. The primary focus of this assessment is the therapist's ability to assign homework that is appropriate and designed for the patient. The therapist should relate these homework assignments to the topic of the session and its general schedule to justify the task at hand. The aim is to jointly select activities that correspond with the therapy session, making sure that the patient understands the objective, to encouraging testing of different ideas and actions while handling any challenges that arise. The therapist should continuously monitor the patient's behavior and offer support.

**To evaluate item 12- homework setting, the following checklist needs to be filled out before scoring this item.**

1. Did the therapist adequately explain the rationale underpinning the assignment?
(A. Yes	B. No	C. Somehow)
2. Did the therapist check that the patient was confident about conducting the task correctly?

(A. Yes	B. No	C. Somehow)

3. Did the patient see the relevance of the assignment?
(A. Yes	B. No)
4. Was the assignment adequately planned within the session?

(A. Yes	B. No)

5. Were the obstacles to conducting the plan discussed?

(A. Yes	B. No)

6. Was the homework task explained sufficiently?
(A. Yes	B. No	C. Somehow)

**Based on this checklist, now encircle the scale the level to which you think the therapist has fulfilled the core function of homework settings. Keep in mind these three aspects of this item:**

(i) Presence/absence of a homework task in which clear and precise goals have been set;
(ii) The task derived from material discussed in the session, such that there is a clear understanding of what was learned from performing the task;
(iii) The homework task was set jointly, and sufficient time was allowed for it to be explained clearly (i.e. Explain, discuss relevance, predict obstacles, etc.).

**Mark or Circle on the vertical line/Scale, the level to which you think the therapist has fulfilled the core function.**
